# Supplementary material for: Digitally predicting protein localization and manipulating protein activity in fluorescence images using 4D reslicing GAN
Source: Bioinformatics. 2022 Nov 14;39(1):btac719. doi: 10.1093/bioinformatics/btac719 (PMC9805574; doi:10.1093/bioinformatics/btac719)
Supplement: btac719_Supplementary_Data [file btac719_supplementary_data.zip › Sample.pdf]

Bioinformatics, YYYY, 0–0
doi: 10.1093/bioinformatics/xxxxx
Advance Access Publication Date: DD Month YYYY
Manuscript Category

Subject Section

Manuscript Title

Corresponding Author[1,*], Co-author[2] and Co-Author[2]

[1]Department of XXXXXXX, Address XXXX etc., [2]Department of XXXXXXX, Address XXXX etc.

*To whom correspondence should be addressed.

Associate Editor: XXXXXXX

Received on XXXXX; revised on XXXXX; accepted on XXXXX

Abstract

Motivation: The quick brown fox jumps over the lazy dog. The quick brown fox jumps over the lazy dog. The quick brown fox jumps over the lazy dog. The quick brown fox jumps over the lazy dog. The quick brown fox jumps over the lazy dog. The quick brown fox jumps over the lazy dog.
Results: The quick brown fox jumps over the lazy dog. The quick brown fox jumps over the lazy dog. The quick brown fox jumps over the lazy dog. The quick brown fox jumps over the lazy dog. The quick brown fox jumps over the lazy dog. The quick brown fox jumps over the lazy dog.
Availability: The quick brown fox jumps over the lazy dog.
Contact: example@example.org
Supplementary information: Supplementary data are available at *Bioinformatics* online.

1 Introduction

The quick brown fox jumps over the lazy dog. The quick brown fox jumps over the lazy dog. The quick brown fox jumps over the lazy dog. The quick brown fox jumps over the lazy dog. The quick brown fox jumps over the lazy dog. The quick brown fox jumps over the lazy dog. The quick brown fox jumps over the lazy dog. The quick brown fox jumps over the lazy dog. The quick brown fox jumps over the lazy dog. The quick brown fox jumps over the lazy dog. The quick brown fox jumps over the lazy dog. The quick brown fox jumps over the lazy dog. The quick brown fox jumps over the lazy dog. The quick brown fox jumps over the lazy dog. The quick brown fox jumps over the lazy dog. The quick brown fox jumps over the lazy dog..

The quick brown fox jumps over the lazy dog. The quick brown fox jumps over the lazy dog.

2 Methods

The quick brown fox jumps over the lazy dog. The quick brown fox jumps over the lazy dog. The quick brown fox jumps over the lazy dog. The quick brown fox jumps over the lazy dog. The quick brown fox jumps over the lazy dog. The quick brown fox jumps over the lazy dog. The quick brown fox jumps over the lazy dog. The quick brown fox jumps over the lazy dog.

The quick brown fox jumps over the lazy dog. The quick brown fox jumps over the lazy dog. The quick brown fox jumps over the lazy dog.

The quick brown fox jumps over the lazy dog. The quick brown fox jumps over the lazy dog. The quick brown fox jumps over the lazy dog. The quick brown fox jumps over the lazy dog. The quick brown fox jumps over the lazy dog. The quick brown fox jumps over the lazy dog. The quick brown fox jumps over the lazy dog. The quick brown fox jumps over the lazy dog. The quick brown fox jumps over the lazy dog. The quick brown fox jumps over the lazy dog. The quick brown fox jumps over the lazy dog. The quick brown fox jumps over the lazy dog. The quick brown fox jumps over the lazy dog. The quick brown fox jumps over the lazy dog. The quick brown fox jumps over the lazy dog. The quick brown fox jumps over the lazy dog. The quick brown fox jumps over the lazy dog.

3 Results

The quick brown fox jumps over the lazy dog. The quick brown fox jumps over the lazy dog.

3.1 Data Structure This is Heading 2 style this is heading 2 style

The quick brown fox jumps over the lazy dog. The quick brown fox jumps over the lazy dog. The quick brown fox jumps over the lazy dog. The quick brown fox jumps over the lazy dog. The quick brown fox jumps over the lazy dog. The quick brown fox jumps over the lazy dog. The quick brown fox jumps over the lazy dog. The quick brown fox jumps over the lazy dog. The quick brown fox jumps over the lazy dog. The quick brown fox jumps over the lazy dog. The quick brown fox jumps over the lazy dog. The quick brown fox jumps over the lazy dog.

3.1.1 This is heading 3 style

The quick brown fox jumps over the lazy dog. The quick brown fox jumps over the lazy dog. The quick brown fox jumps over the lazy dog. The quick brown fox jumps over the lazy dog.

(1) The quick brown fox jumps over the lazy dog. The quick brown fox jumps over the lazy dog.

(2) The quick brown fox jumps over the lazy dog. The quick brown fox jumps over the lazy dog.

(3) The quick brown fox jumps over the lazy dog.

(4) The quick brown fox jumps over the lazy dog. The quick brown fox jumps over the lazy dog.

(5) The quick brown fox jumps over the lazy dog. The quick brown fox jumps over the lazy dog.

(6) The quick brown fox jumps over the lazy dog. The quick brown fox jumps over the lazy dog.

The quick brown fox jumps over the lazy dog. The quick brown fox jumps over the lazy dog.

- The quick brown fox jumps over the lazy dog. The quick brown fox jumps over the lazy dog.
- The quick brown fox jumps over the lazy dog. The quick brown fox jumps over the lazy dog.
- The quick brown fox jumps over the lazy dog. The quick brown fox jumps over the lazy dog.
- The quick brown fox jumps over the lazy dog. The quick brown fox jumps over the lazy dog.
- The quick brown fox jumps over the lazy dog. The quick brown fox jumps over the lazy dog.

The quick brown fox jumps over the lazy dog. The quick brown fox jumps over the lazy dog. The quick brown fox jumps over the lazy dog.

3.2 Unnumbered list style

The quick brown fox jumps over the lazy dog. The quick brown fox jumps over the lazy dog. The quick brown fox jumps over the lazy dog.

The quick brown fox jumps over the lazy dog. The quick brown fox jumps over the lazy dog.

The quick brown fox jumps over the lazy dog. The quick brown fox jumps over the lazy dog.

The quick brown fox jumps over the lazy dog. The quick brown fox jumps over the lazy dog.

The quick brown fox jumps over the lazy dog. The quick brown fox jumps over the lazy dog.

The quick brown fox jumps over the lazy dog. The quick brown fox jumps over the lazy dog.

$$Pr(\mu) = a_\mu \Big/ \sum_j a_j \tag{1}$$

The quick brown fox jumps over the lazy dog. The quick brown fox jumps over the lazy dog. The quick brown fox jumps over the lazy dog. The quick brown fox jumps over the lazy dog. The quick brown fox jumps over the lazy dog.

Fig. 1. Relation between τ and *t*. This example has only two continuous Steppers, S_1 and S_2.

The quick brown fox jumps over the lazy dog. The quick brown fox jumps over the lazy dog. The quick brown fox jumps over the lazy dog. The quick brown fox jumps over the lazy dog. The quick brown fox jumps over the lazy dog. The quick brown fox jumps over the lazy dog. The quick brown fox jumps over the lazy dog. The quick brown fox jumps over the lazy dog. The quick brown fox jumps over the lazy dog. The quick brown fox jumps over the lazy dog. The quick brown fox jumps over the lazy dog. The quick brown fox jumps over the lazy dog. The quick brown fox jumps over the lazy dog. The quick brown fox jumps over the lazy dog. The quick brown fox jumps over the lazy dog.

The quick brown fox jumps over the lazy dog. The quick brown fox jumps over the lazy dog. The quick brown fox jumps over the lazy dog. The quick brown fox jumps over the lazy dog. The quick brown fox jumps over the lazy dog. The quick brown fox jumps over the lazy dog. The quick brown fox jumps over the lazy dog. The quick brown fox jumps over the lazy dog. The quick brown fox jumps over the lazy dog. The quick brown fox jumps over the lazy dog. The quick brown fox jumps over the lazy dog. The quick brown fox jumps over the lazy dog. The quick brown fox jumps over the lazy dog. The quick brown fox jumps over the lazy dog. The quick brown fox jumps over the lazy dog.

The quick brown fox jumps over the lazy dog. The quick brown fox jumps over the lazy dog.

Table 1. Benchmark results of the cascade oscillators model

|S|	Predicted cost	Timing	Predicted speed	Speed
1	S219.20(100%)	68m43s	1.00	1.00
2	2^9.10+2^{19}.10(~50%)	35m13s	2.00	1.95
4	2^{19}.20(100%)	68m43s	1.00	1.00
10	2^9.10+2^{19}.10(~50%)	35m13s	2.00	1.95
20	2^{19}.20(100%)	68m43s	1.00	9.5

This is table foot note sample text This is table foot note sample text This is table foot note sample text

The quick brown fox jumps over the lazy dog. The quick brown fox jumps over the lazy dog. The quick brown fox jumps over the lazy dog. The quick brown fox jumps over the lazy dog. The quick brown fox jumps over the lazy dog. The quick brown fox jumps over the lazy dog. The quick brown fox jumps over the lazy dog. The quick brown fox jumps over the lazy dog.The quick brown fox jumps over the lazy dog. The quick brown fox jumps over the lazy dog. The quick brown fox jumps over the lazy dog. The quick brown fox jumps over the lazy dog. The quick brown fox jumps over the lazy dog. The quick brown fox jumps over the lazy dog.The quick brown fox jumps over the lazy dog.

Acknowledgements

The quick brown fox jumps over the lazy dog. The quick brown fox jumps over the lazy dog. The quick brown fox jumps over the lazy dog. The quick brown fox jumps over the lazy dog. The quick brown fox jumps over the lazy dog. The quick brown fox jumps over the lazy dog.

Funding

This work has been supported by the …..

Conflict of Interest: none declared.

References

Alexandrescu,A. (2001) Modern C++ Design: Generic Programming and Design Patterens Applied. Addision Wesley Professional, Boston.

Dormand,J.R. and Prince,P.J. (1980) A family of embedded Runge–Kutta formulae. *J. Comp. Appl. Math.*, **6**, 19–26.

Alexandrescu,A. (2001) *Modern C++ Design: Generic Programming and Design Patterens Applied.* Addision Wesley Professional, Boston.

Dormand,J.R. and Prince,P.J. (1980) A family of embedded Runge–Kutta formulae. *J. Comp. Appl. Math.*, **6**, 19–26.

Alexandrescu,A. (2001) *Modern C++ Design: Generic Programming and Design Patterens Applied.* Addision Wesley Professional, Boston.

Dormand,J.R. and Prince,P.J. (1980) A family of embedded Runge–Kutta formulae. *J. Comp. Appl. Math.*, **6**, 19–26.

Yoo,M.S. *et al*. (2003) Oxidative stress regulated genes in nigral dopaminergic neurnol cell: correlation with the known pathology in Parkinson's disease. *Brain Res. Mol. Brain Res.*, **110**(Suppl. 1), 76–84.

Crenshaw, B.,III, and Jones, W.B.,Jr (2003) The future of clinical cancer management: one tumor, one chip. *Bioinformatics*, doi:10.1093/bioinformatics/btn000.

Auhtor,A.B. *et al.* (2000) Chapter title. In Smith, A.C. (ed.), *Book Title*, 2nd edn. Publisher, Location, Vol. 1, pp. ???–???.

Bardet, G. (1920) Sur un syndrome d'obesite infantile avec polydactylie et retinite pigmentaire (contribution a l'etude des formes cliniques de l'obesite hypophysaire). PhD Thesis, name of institution, Paris, France.
