# Supplementary material for: Digitally predicting protein localization and manipulating protein activity in fluorescence images using 4D reslicing GAN
Source: Bioinformatics. 2022 Nov 14;39(1):btac719. doi: 10.1093/bioinformatics/btac719 (PMC9805574; doi:10.1093/bioinformatics/btac719)
Supplement: btac719_Supplementary_Data [file btac719_supplementary_data.zip › figures/whyMyoJubbad.drawio.pdf]

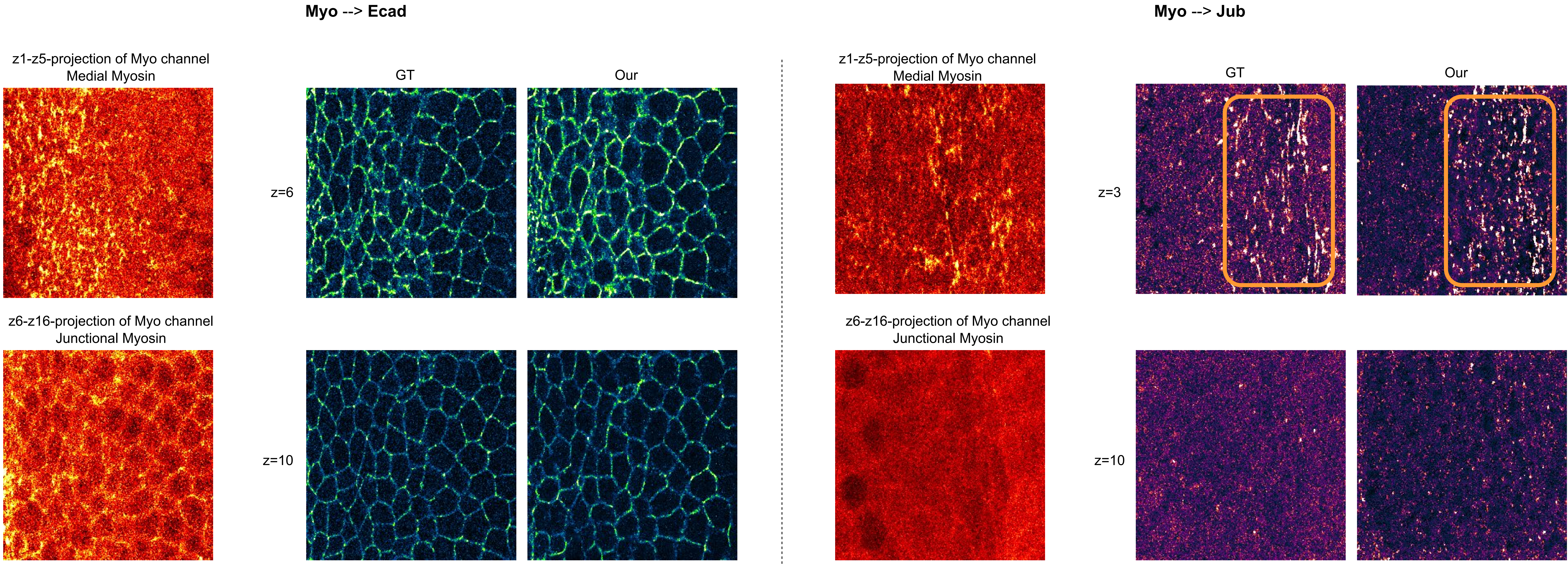
Myo --> Ecad
Myo --> Jub
z1-z5-projection of Myo channel
Medial Myosin
GT
Our
z=6
z6-z16-projection of Myo channel
Junctional Myosin
z=10
z1-z5-projection of Myo channel
Medial Myosin
GT
Our
z=3
z6-z16-projection of Myo channel
Junctional Myosin
z=10
