# Supplementary figures and images for: Digitally predicting protein localization and manipulating protein activity in fluorescence images using 4D reslicing GAN
Source: Bioinformatics. 2022 Nov 14;39(1):btac719. doi: 10.1093/bioinformatics/btac719 (PMC9805574; doi:10.1093/bioinformatics/btac719)

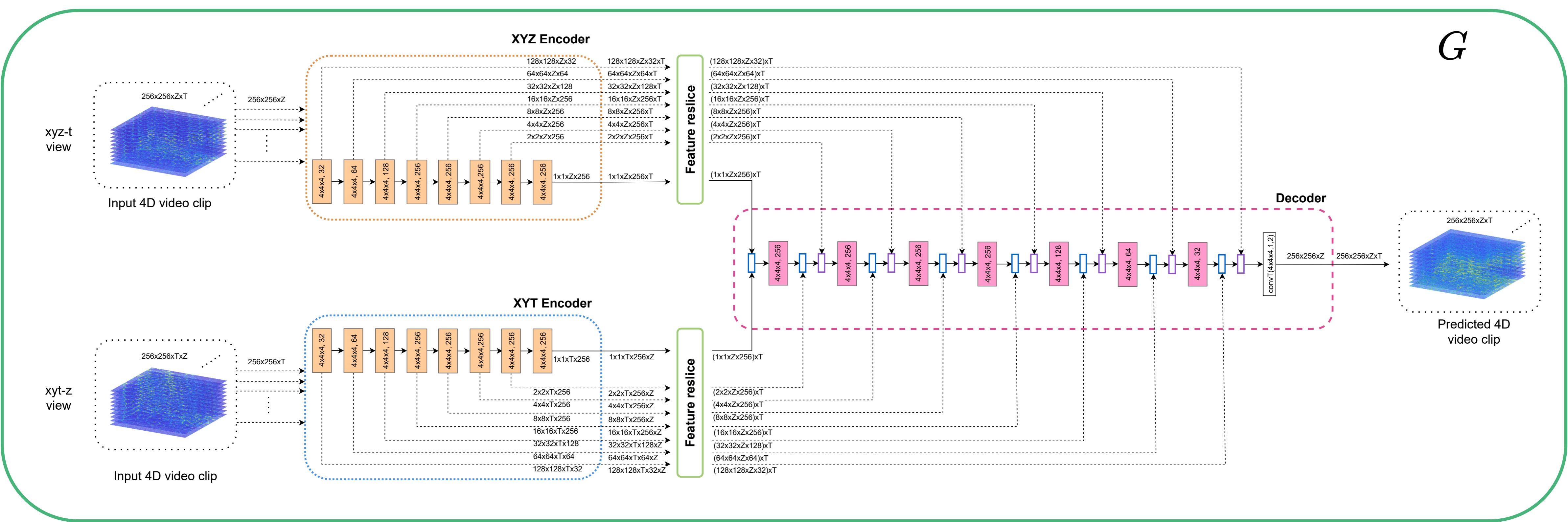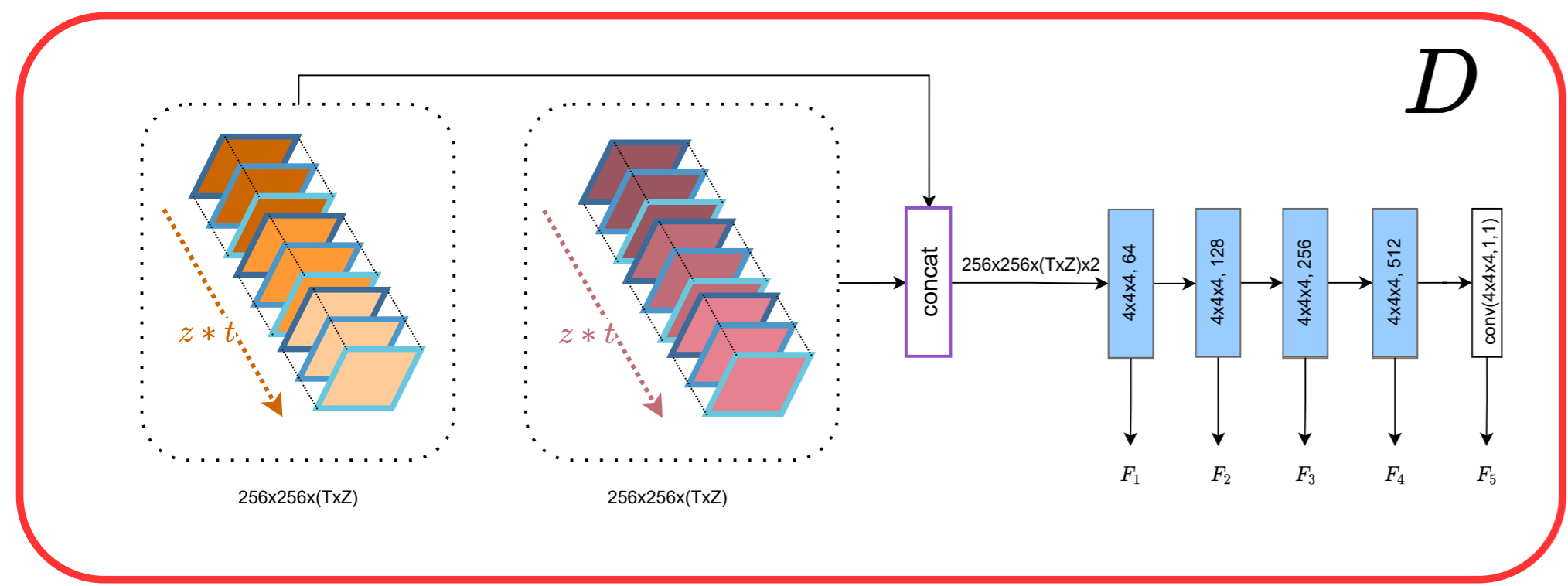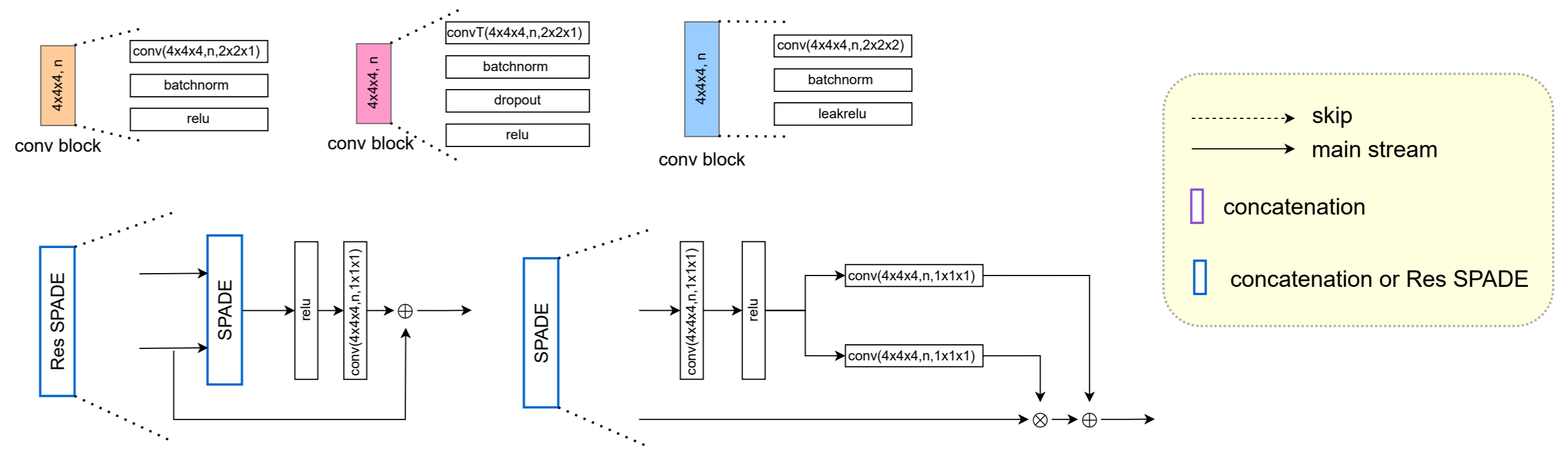

Supplement: btac719_Supplementary_Data [file btac719_supplementary_data.zip › figures/figure_method1_1.drawio.pdf]

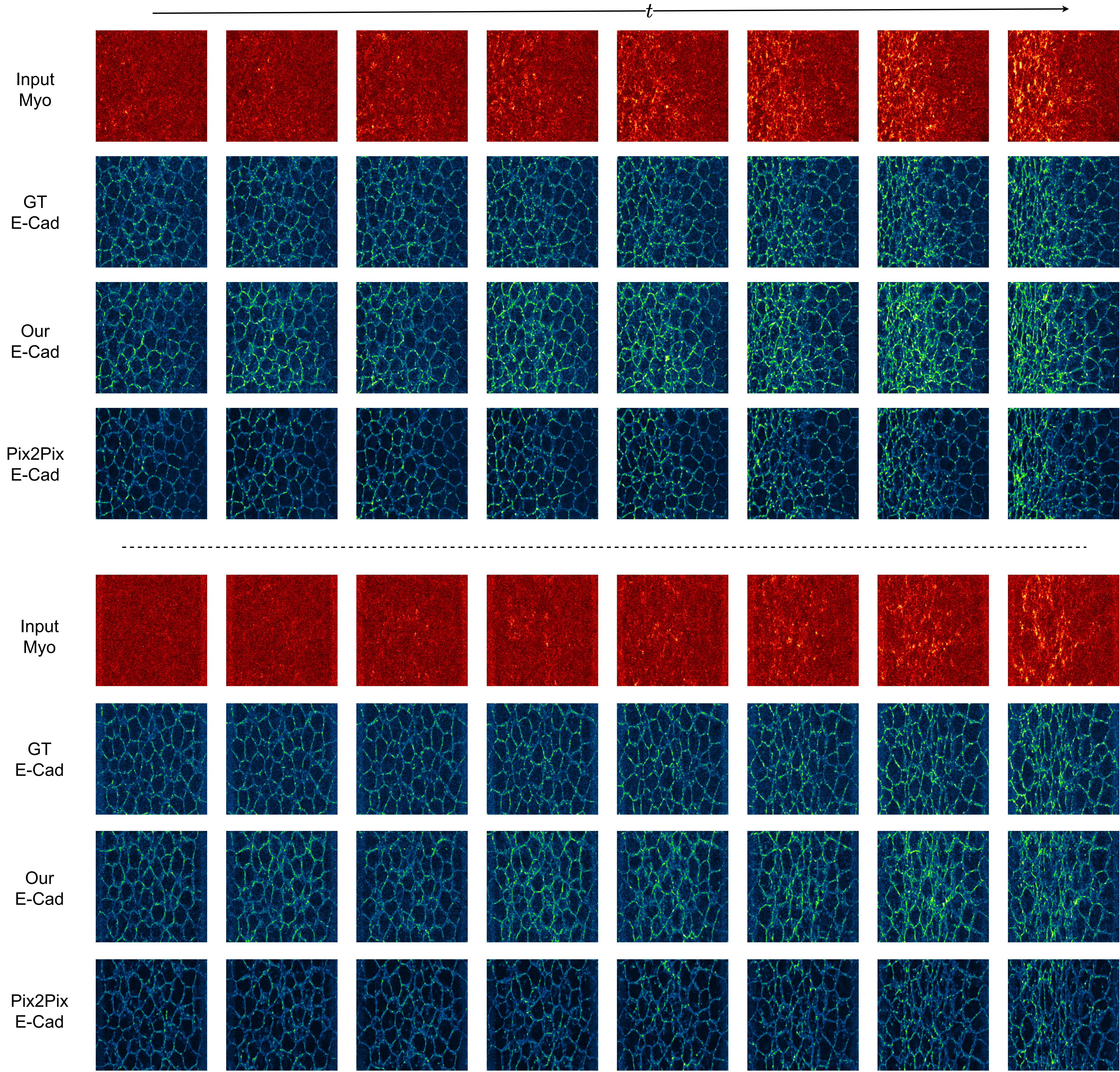

Supplement: btac719_Supplementary_Data [file btac719_supplementary_data.zip › figures/spp_more_PLP_myo-ecad.drawio.pdf]

## Digital Inactivation (DI)

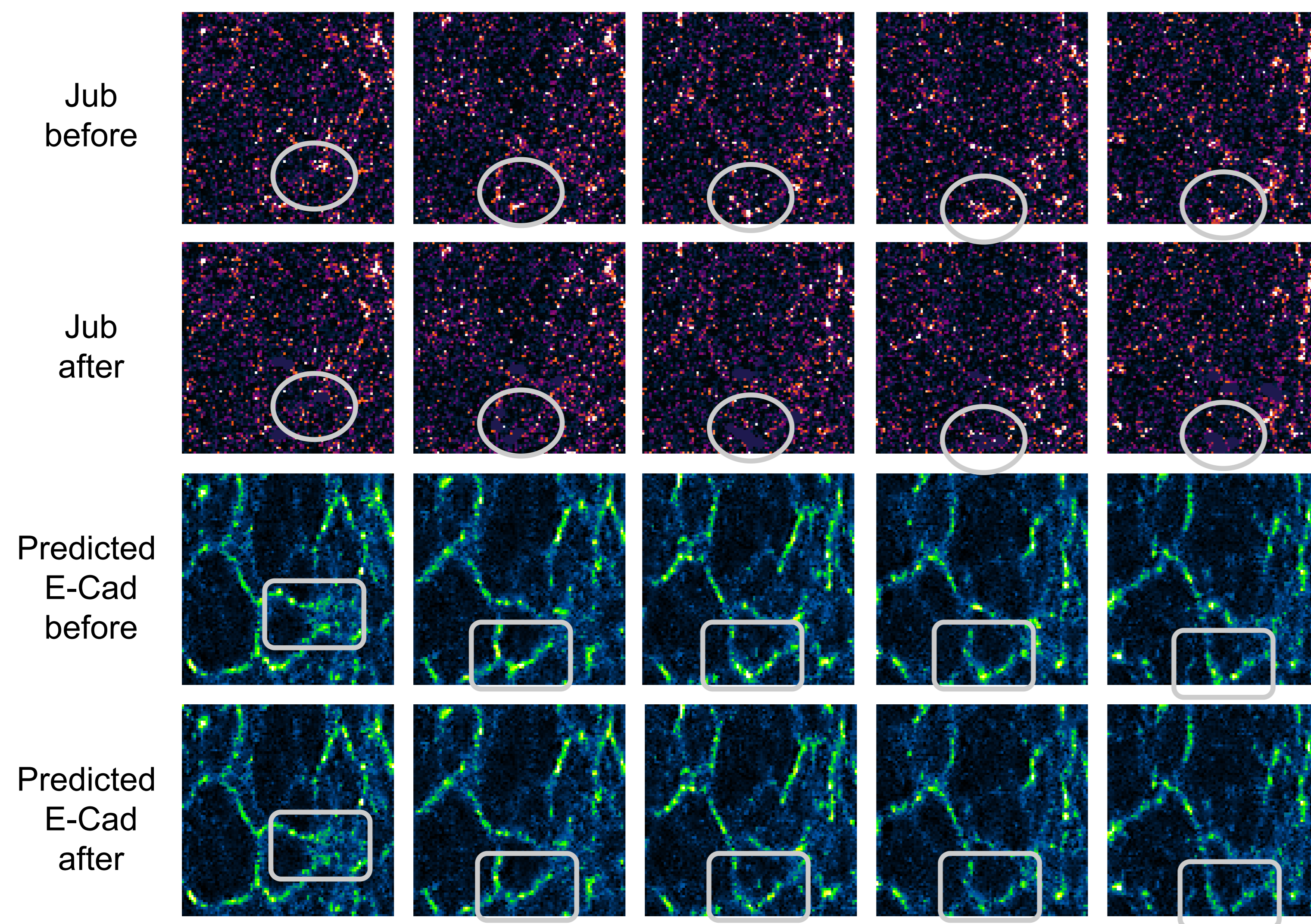

## Digital Activation (DA)

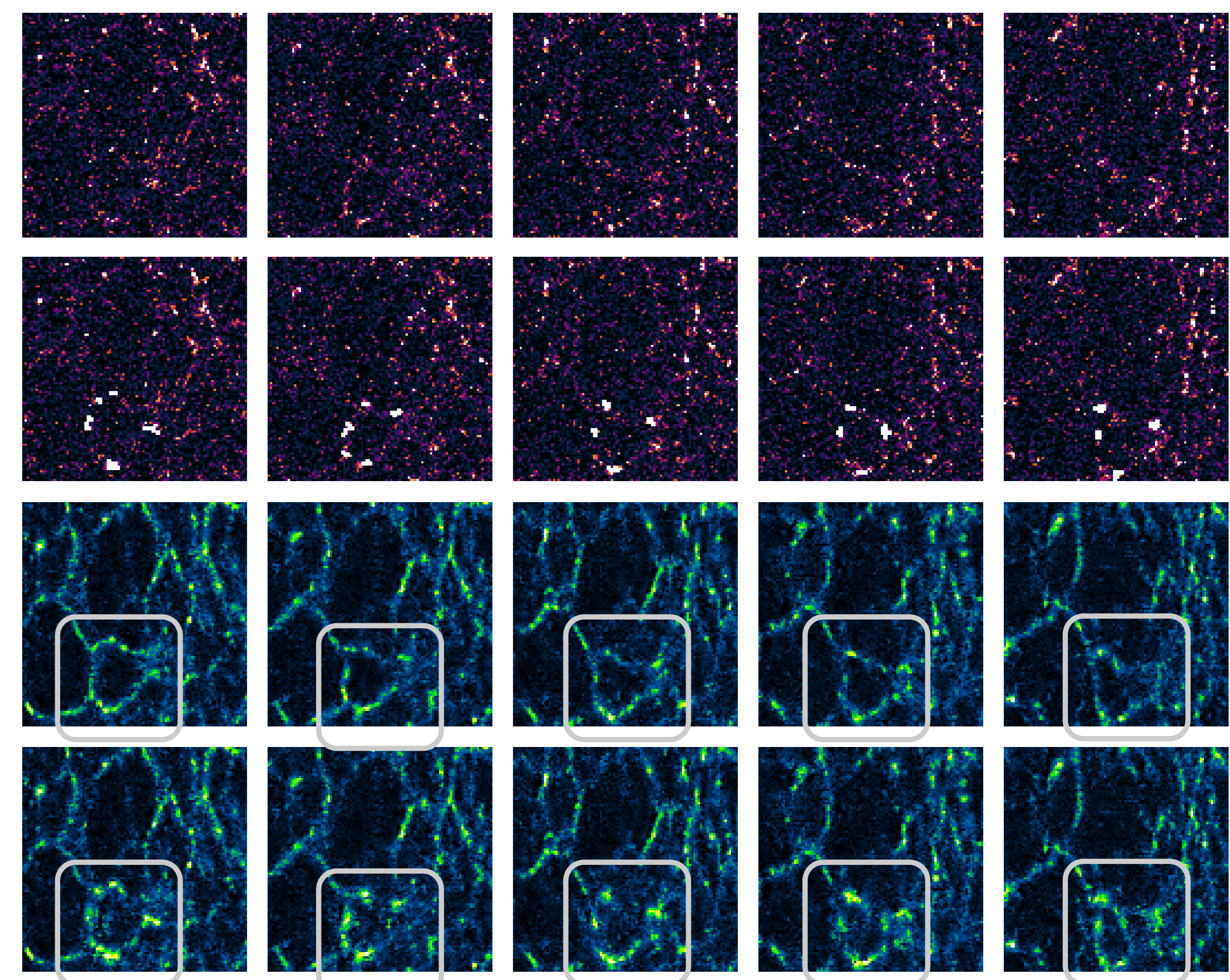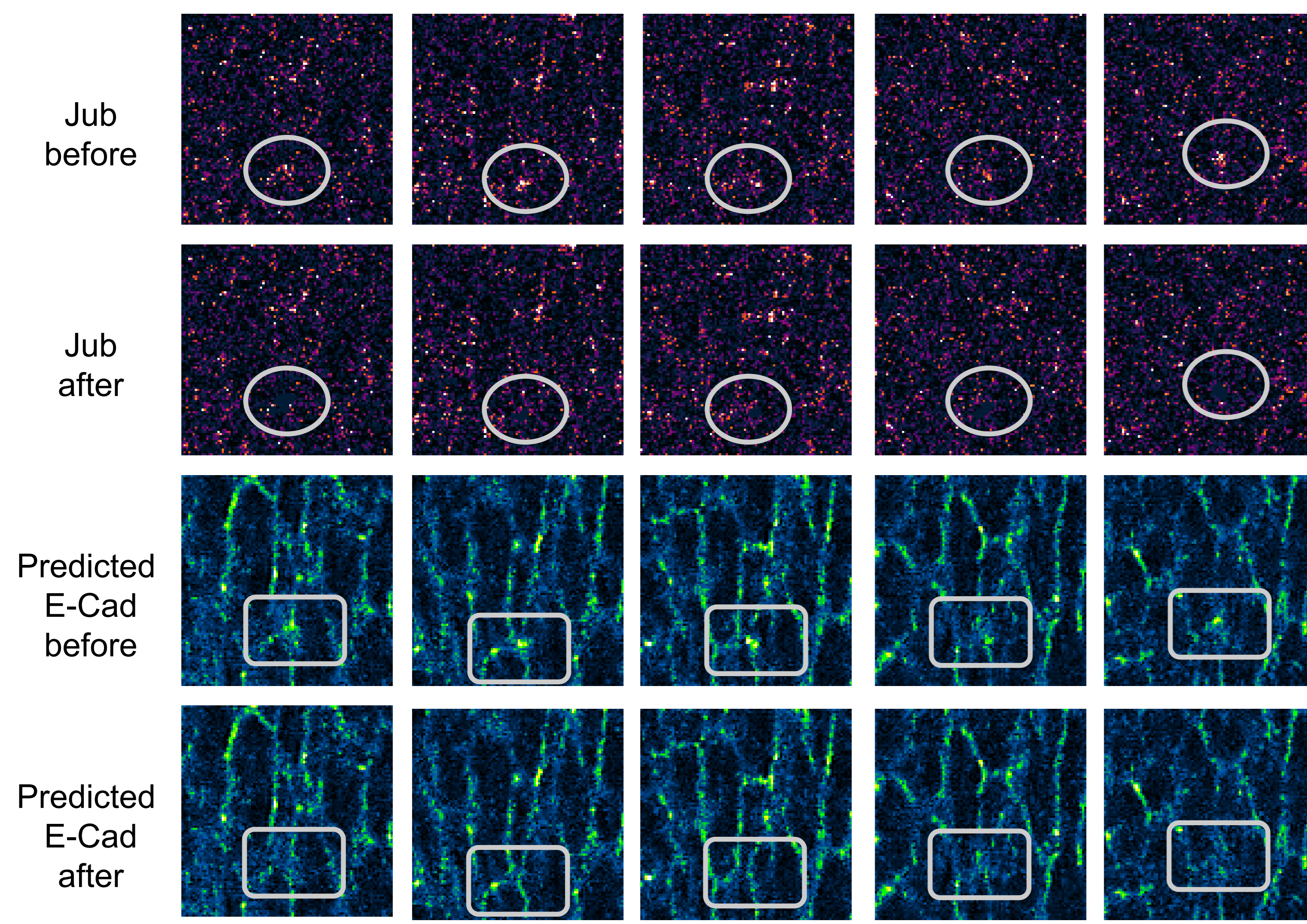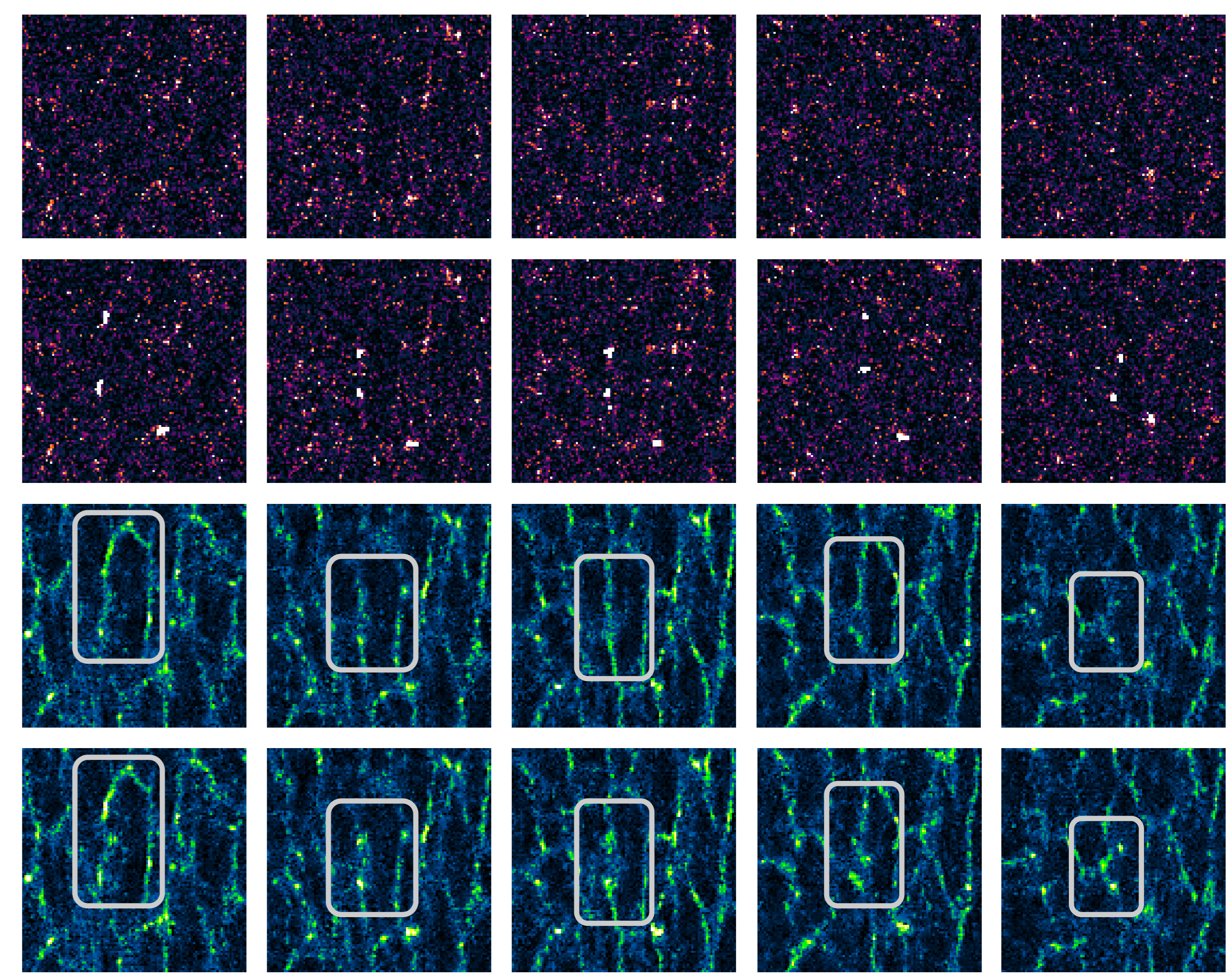

Supplement: btac719_Supplementary_Data [file btac719_supplementary_data.zip › figures/spp_more_DK_aju-ecad.drawio.pdf]

## Digital Inactivation (DI)

## Digital Activation (DA)

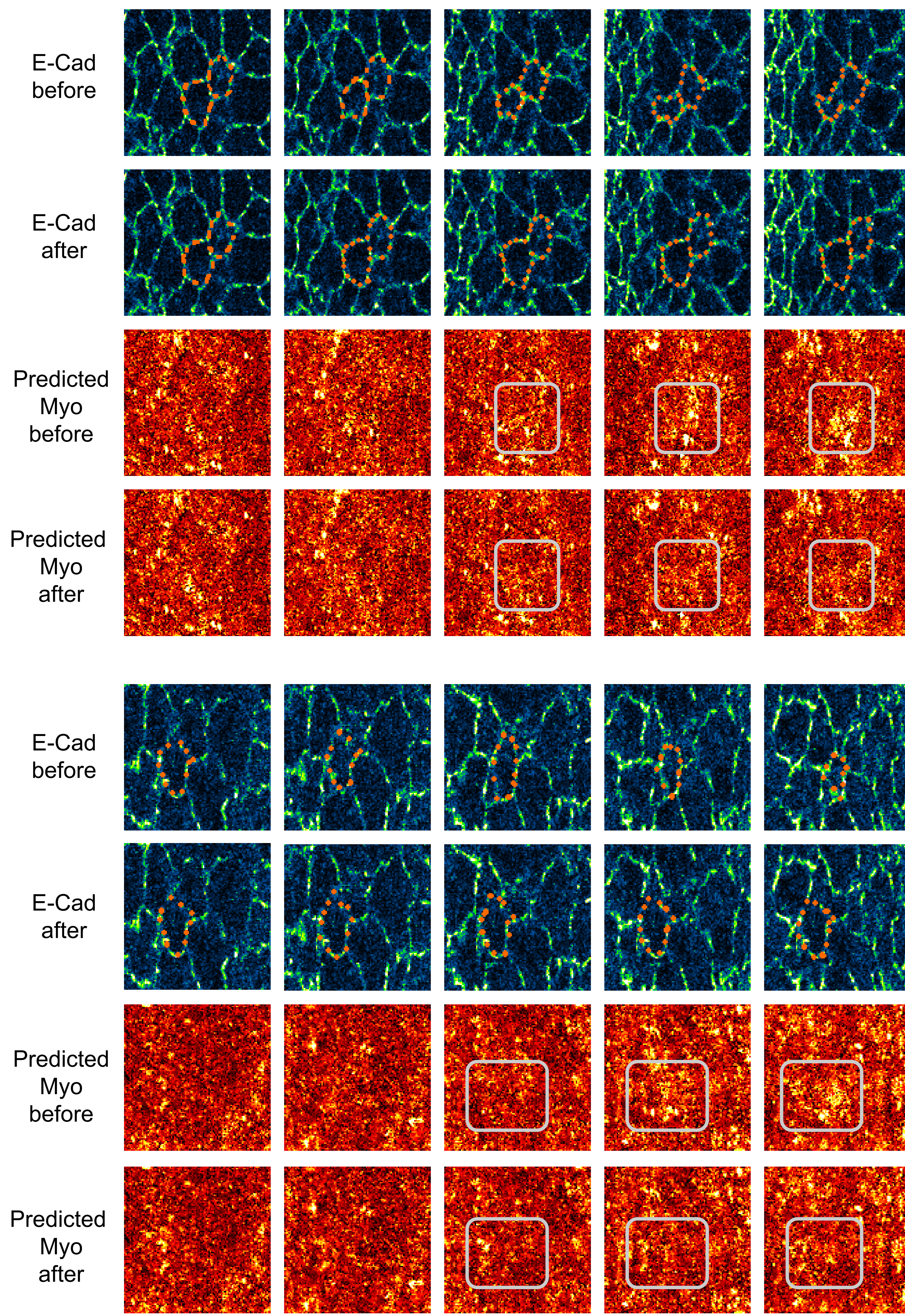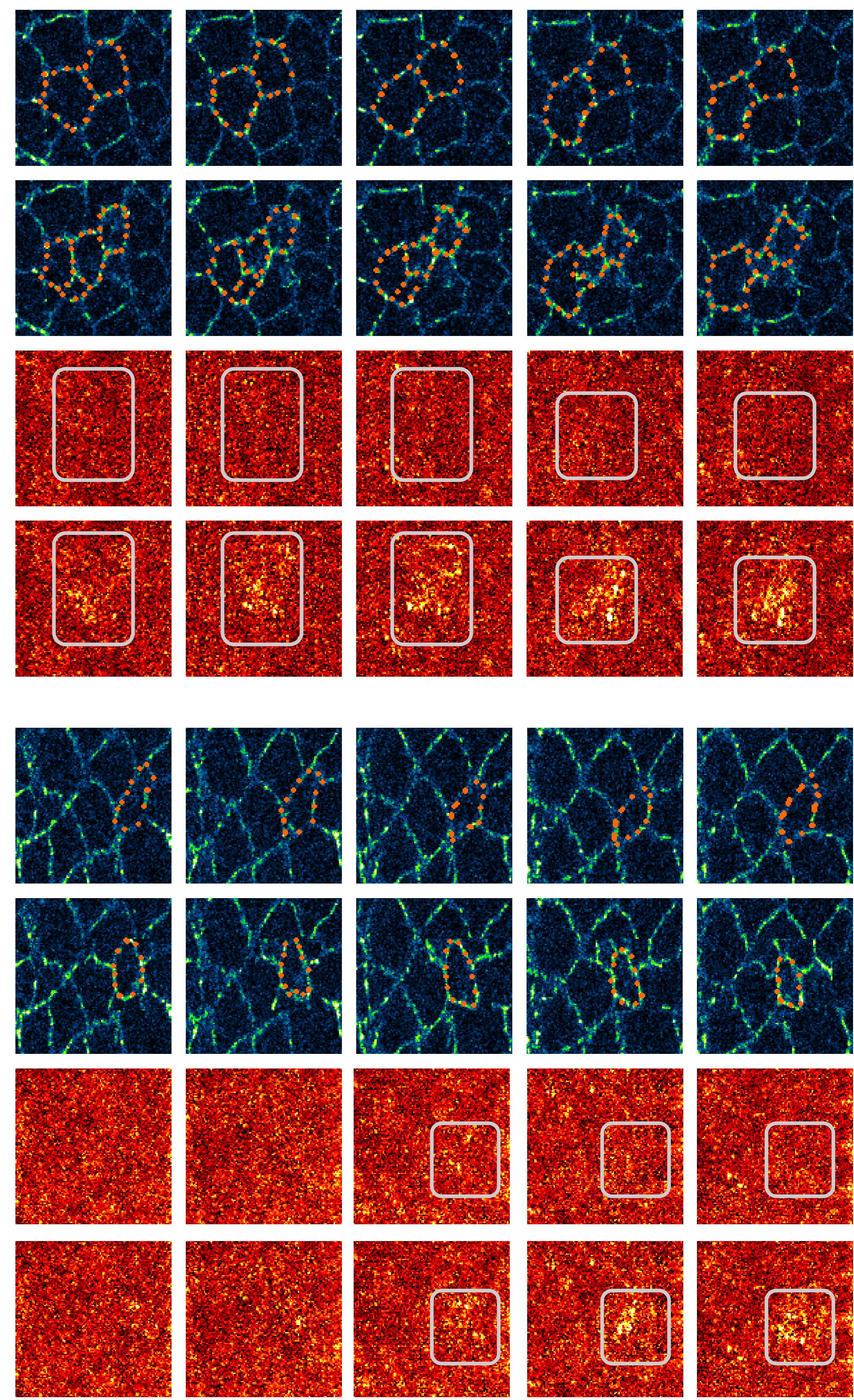

Supplement: btac719_Supplementary_Data [file btac719_supplementary_data.zip › figures/spp_more_DK_ecad-myo.drawio.pdf]

## Digital Inactivation (DI)

## Digital Activation (DA)

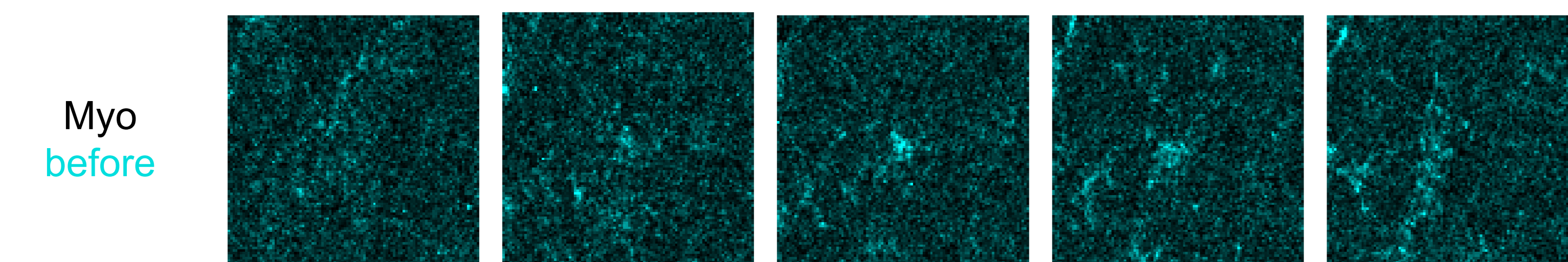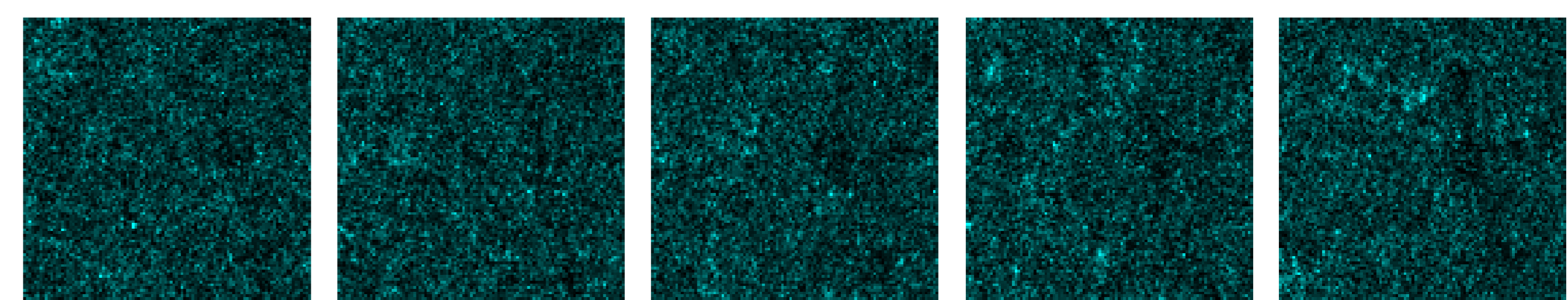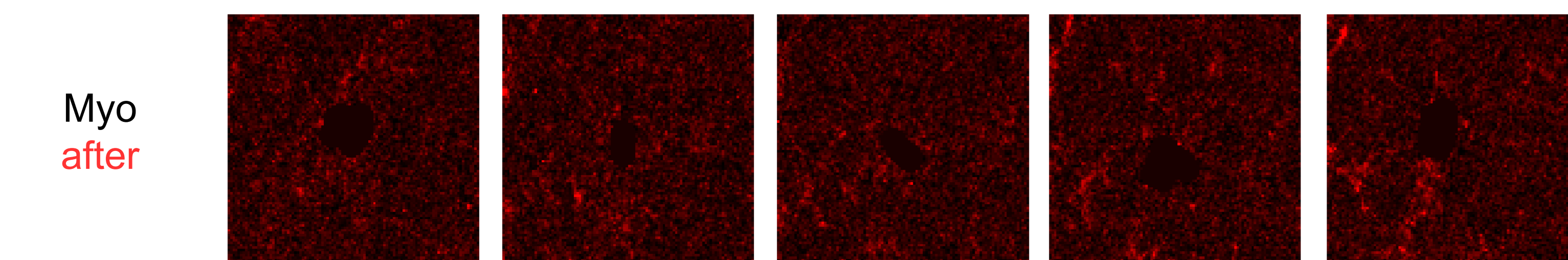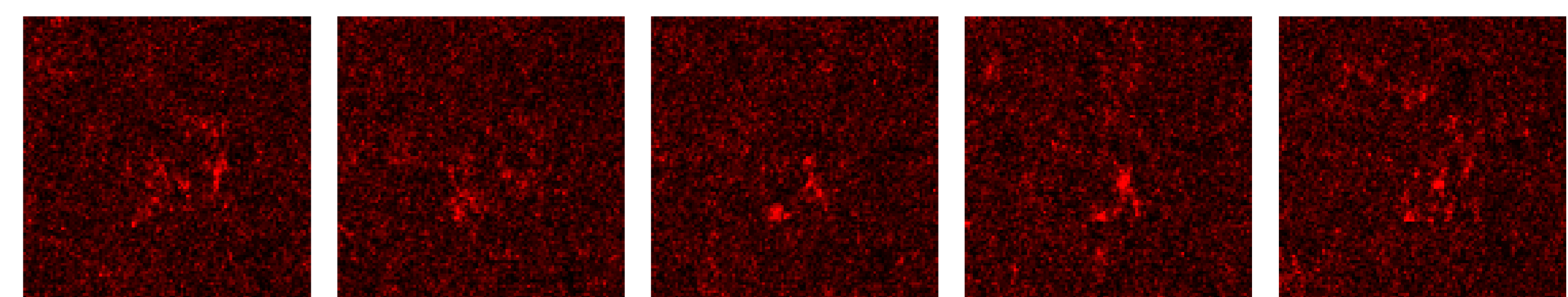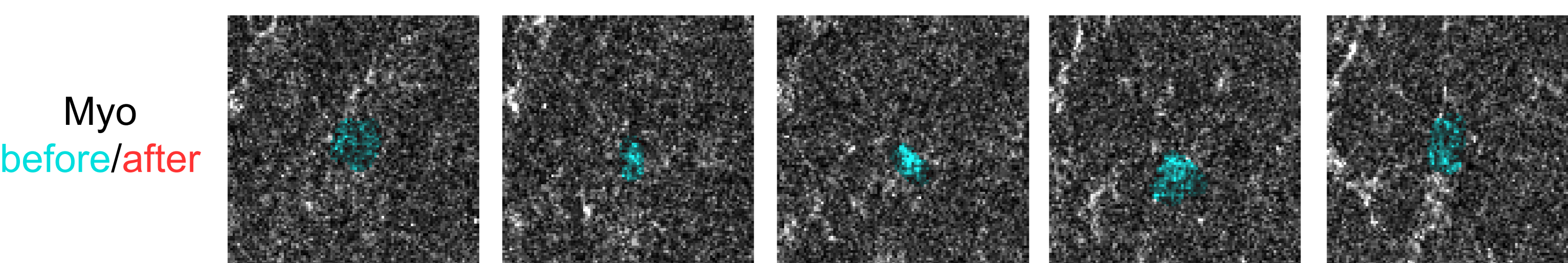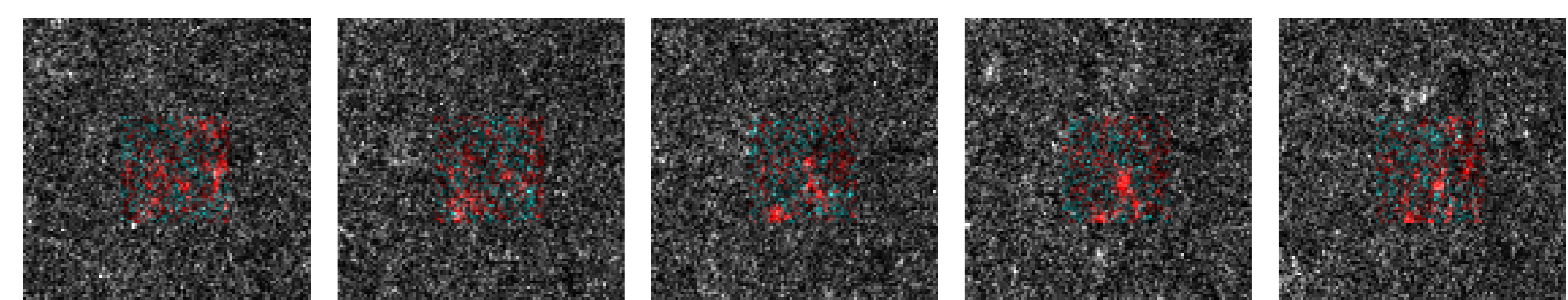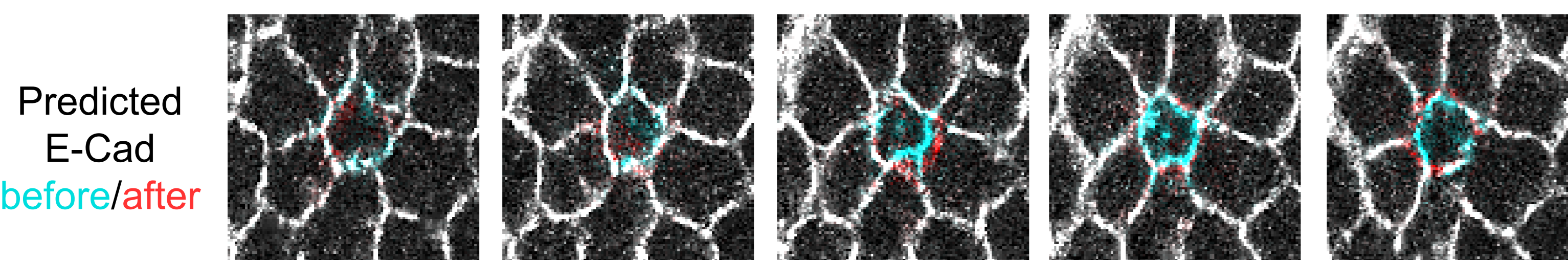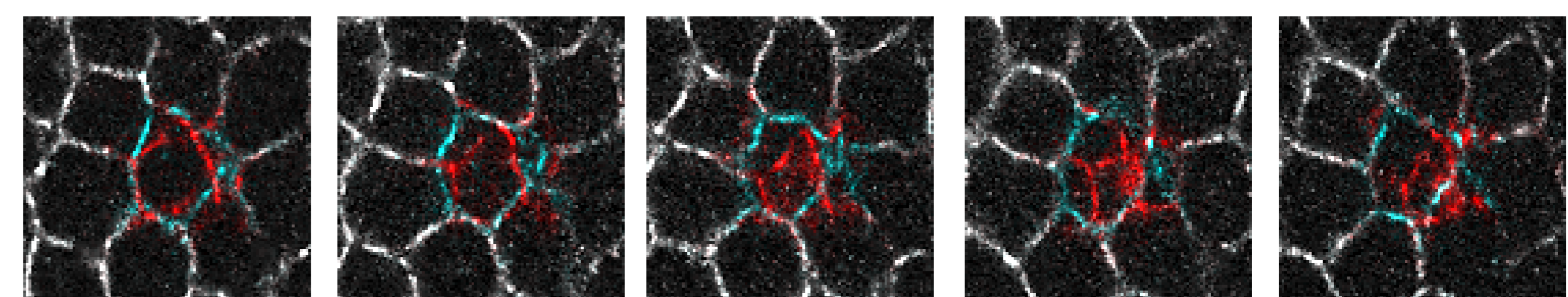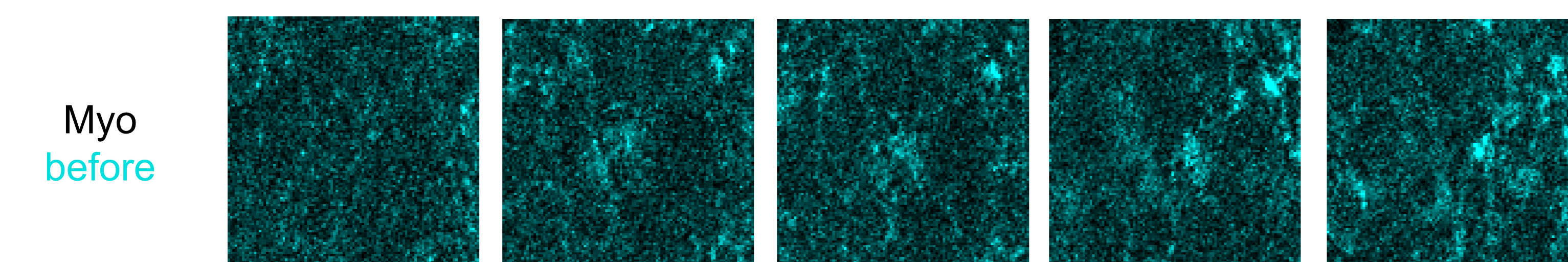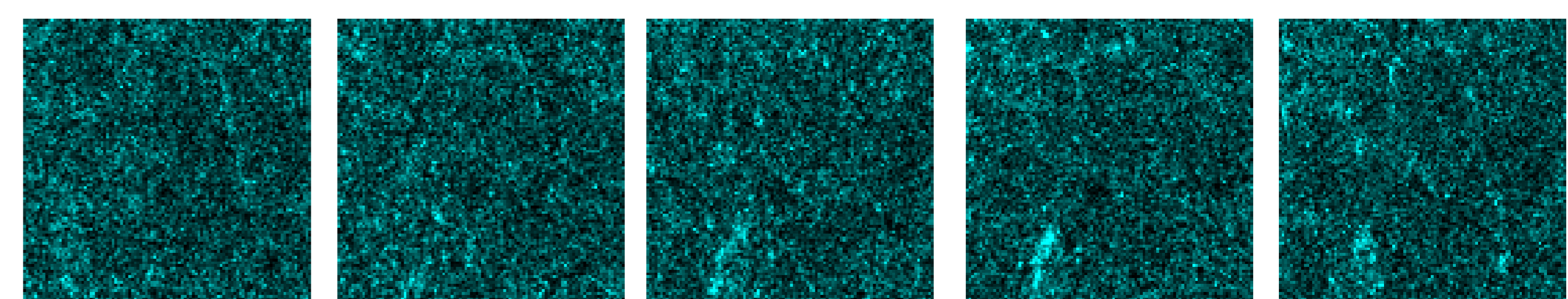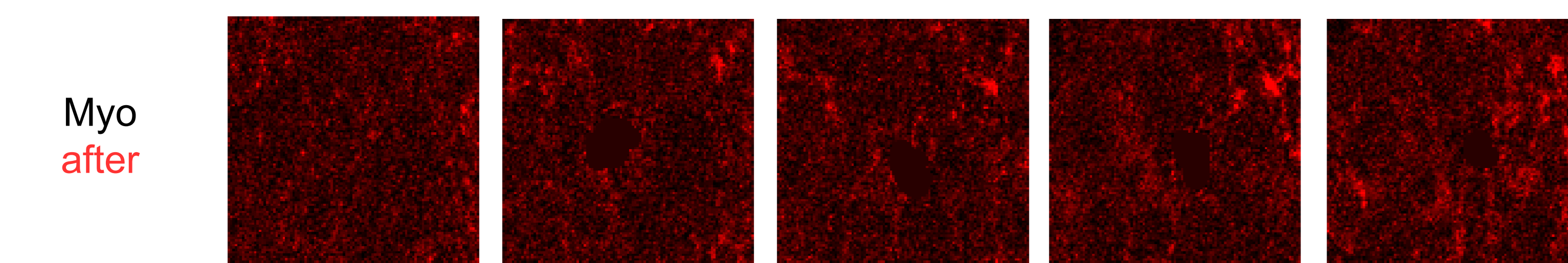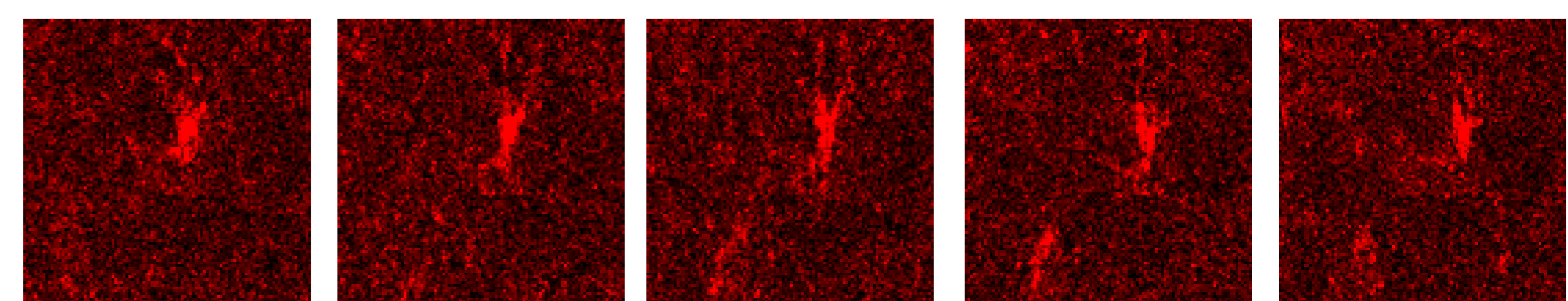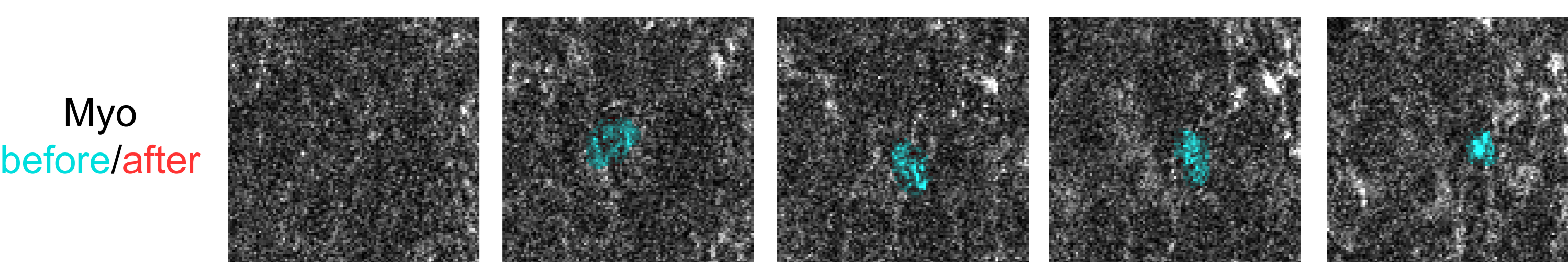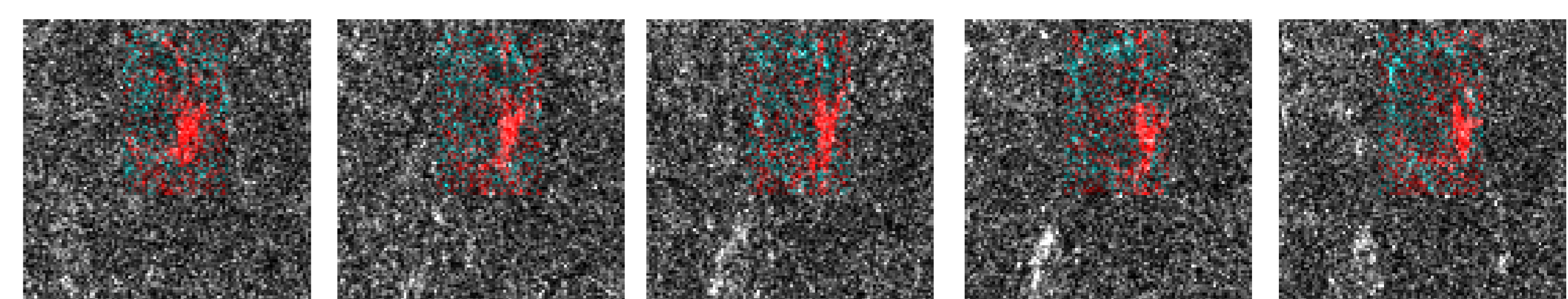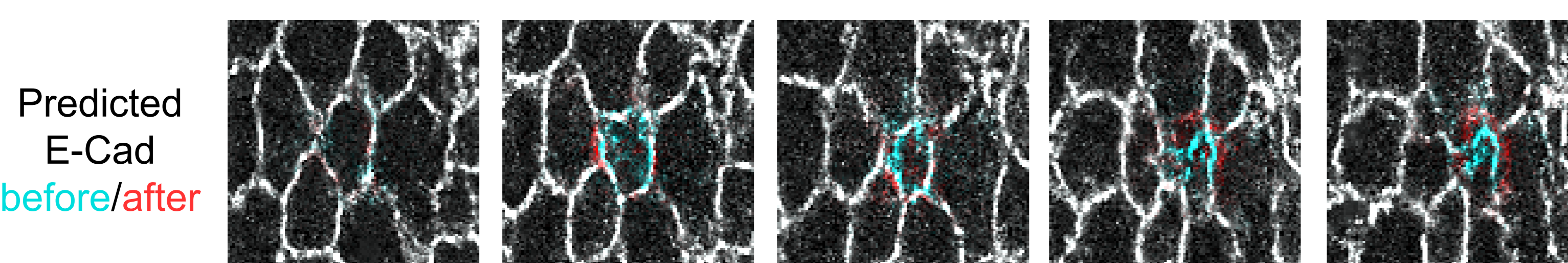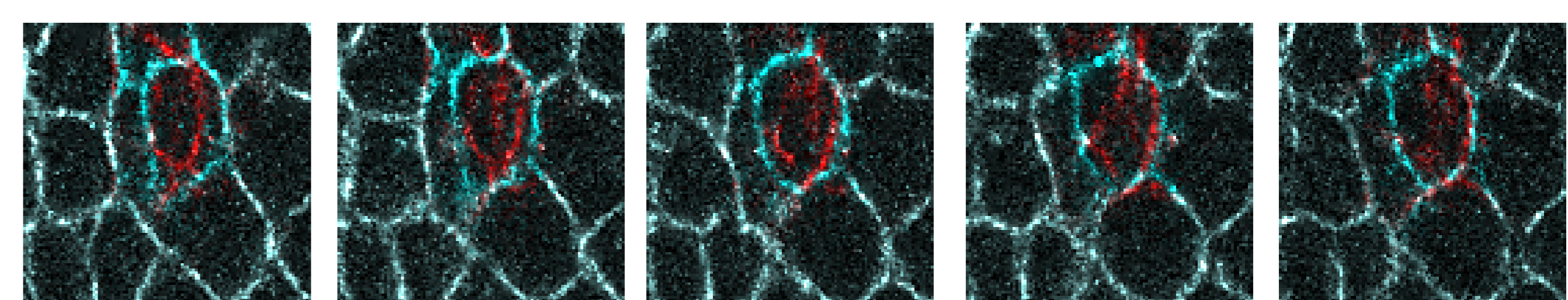

Supplement: btac719_Supplementary_Data [file btac719_supplementary_data.zip › figures/spp_more_DK_myo-ecad.drawio.pdf]

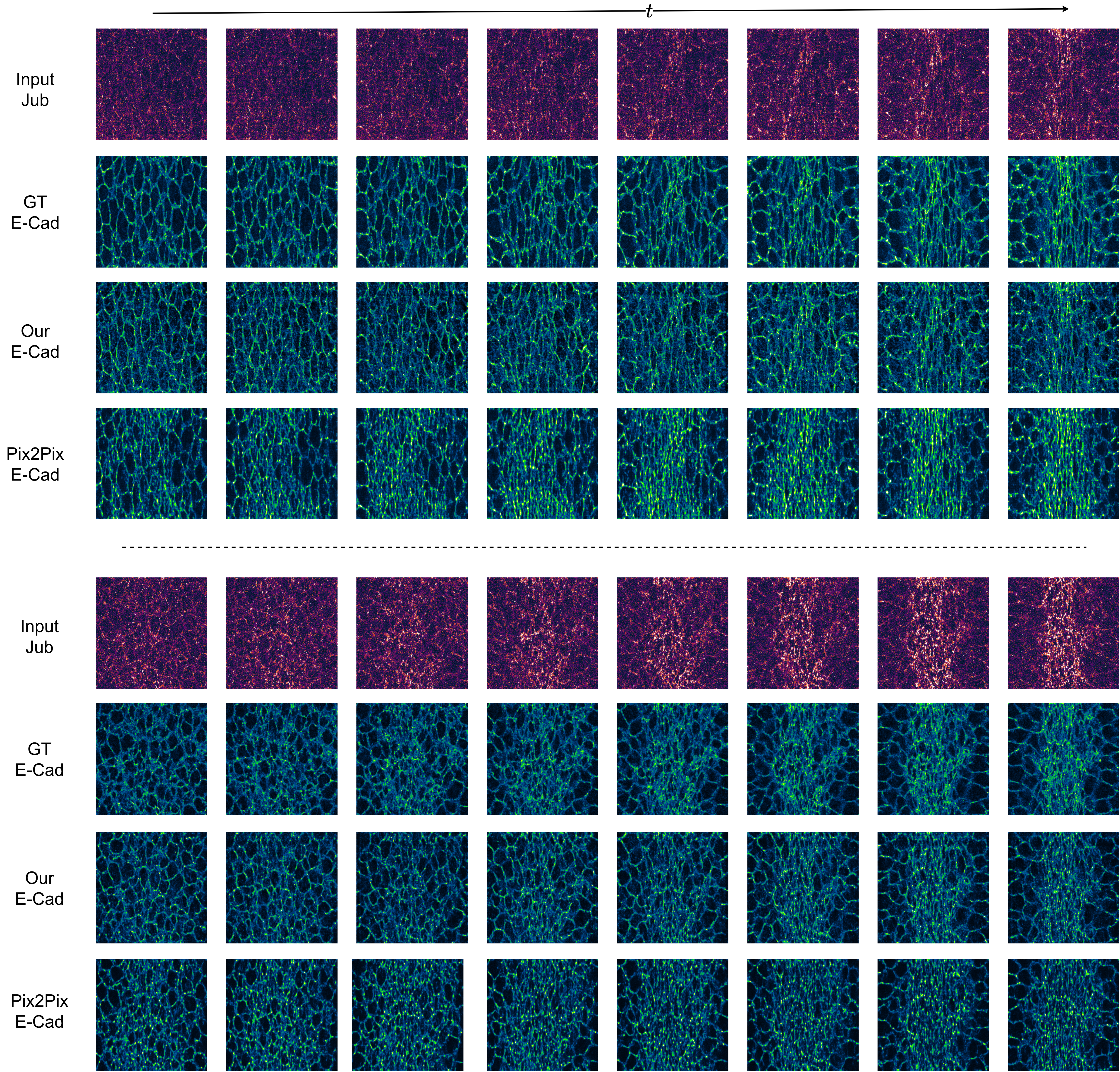

Supplement: btac719_Supplementary_Data [file btac719_supplementary_data.zip › figures/spp_more_PLP_aju-ecad.drawio.pdf]

$t$ 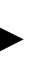Input  
Jub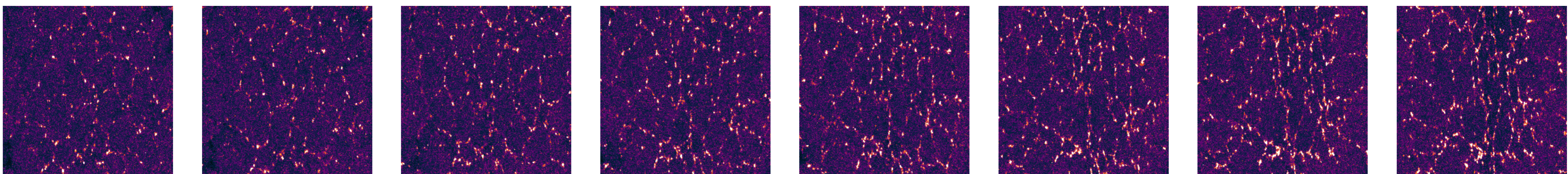GT  
Myo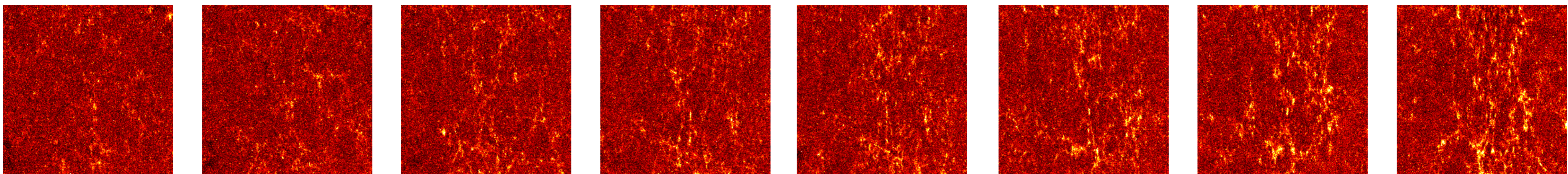Our  
Myo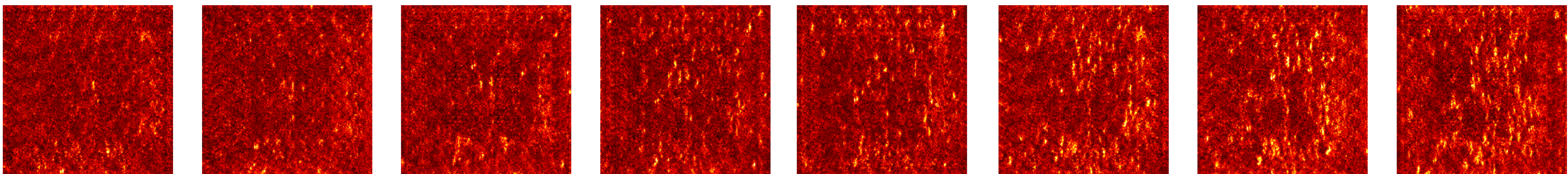Pix2Pix  
Myo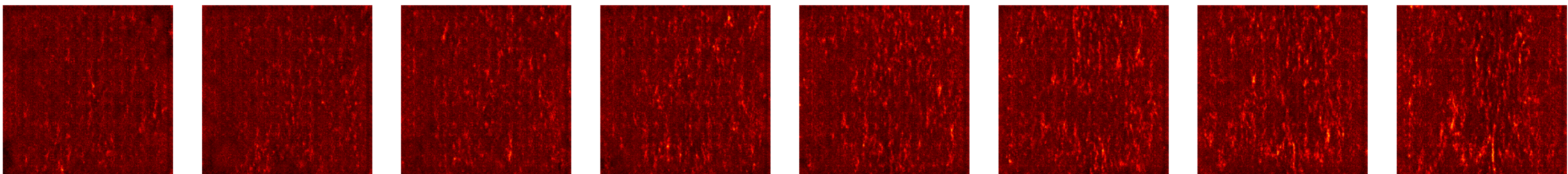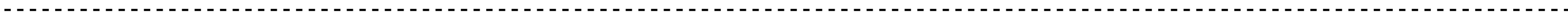Input  
Jub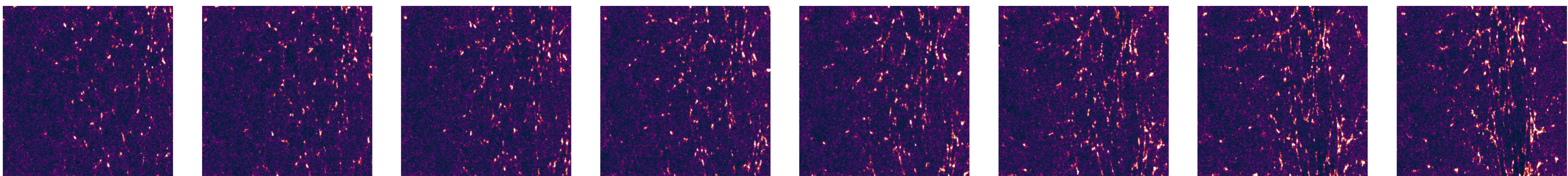GT  
Myo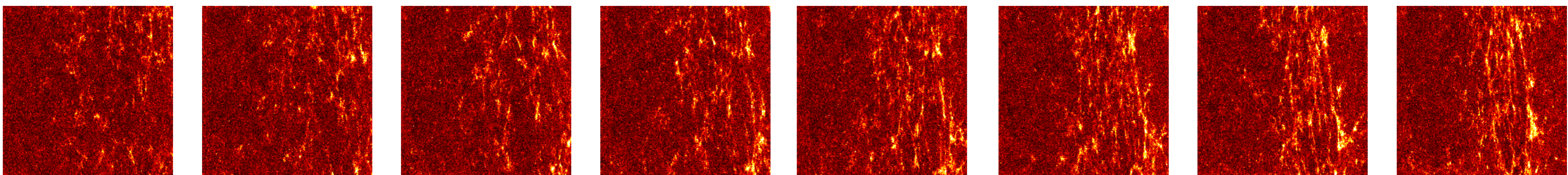Our  
Myo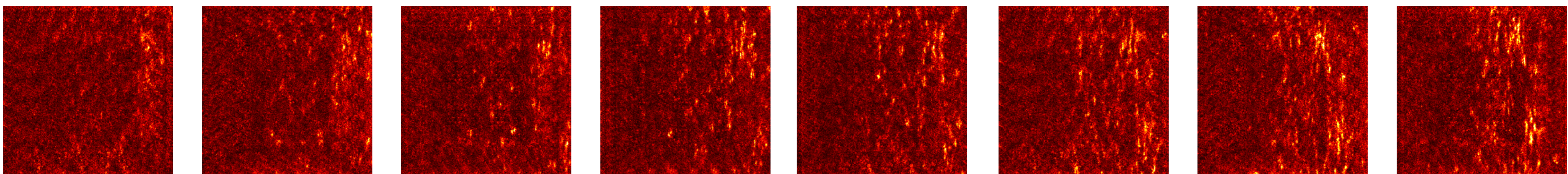Pix2Pix  
Myo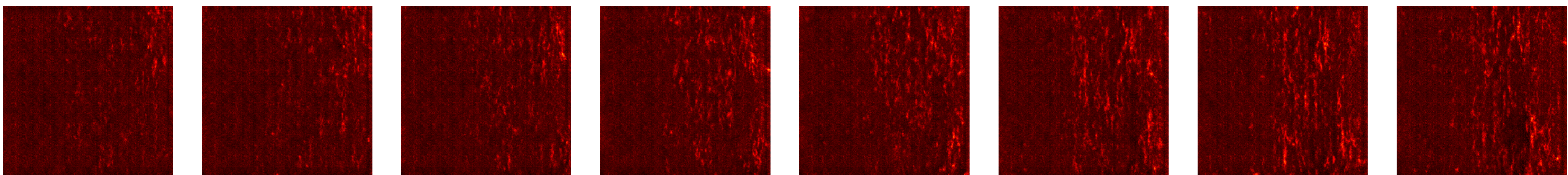

Supplement: btac719_Supplementary_Data [file btac719_supplementary_data.zip › figures/spp_more_PLP_aju-myo.drawio.pdf]

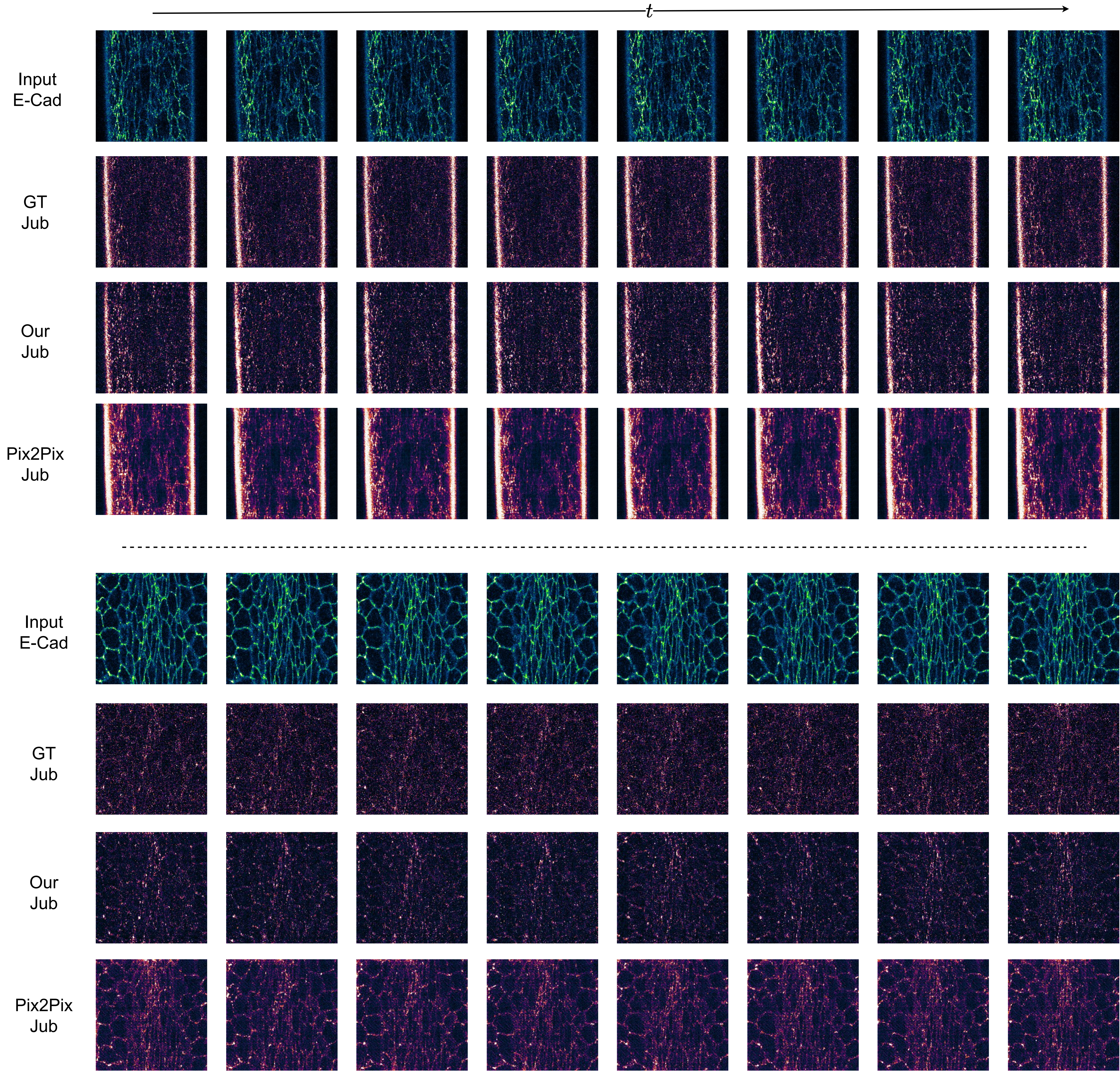

Supplement: btac719_Supplementary_Data [file btac719_supplementary_data.zip › figures/spp_more_PLP_ecad-aju.drawio.pdf]

$t$

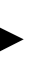

Input  
Myo

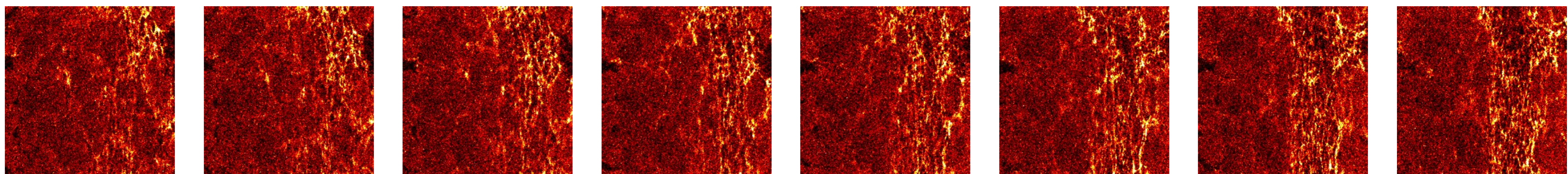

GT  
Jub

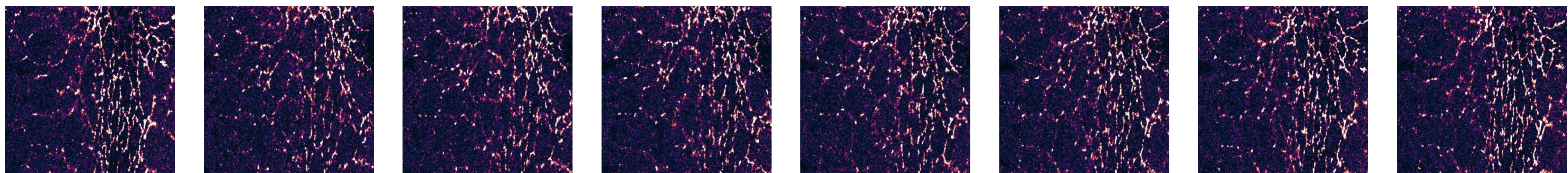

Our  
Jub

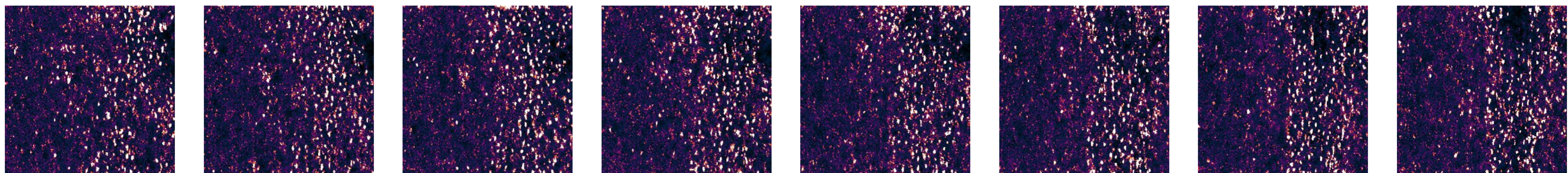

Pix2Pix  
Jub

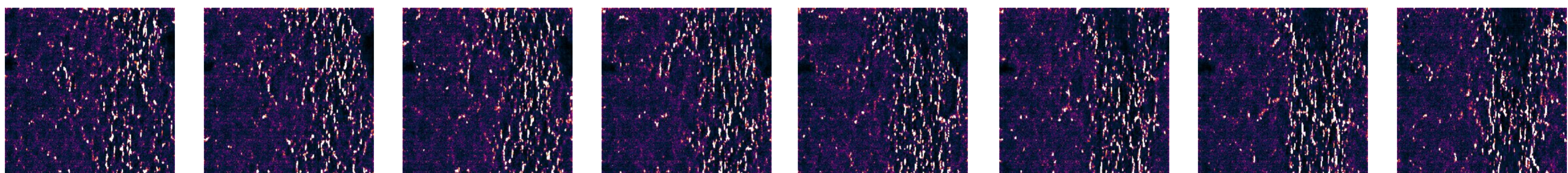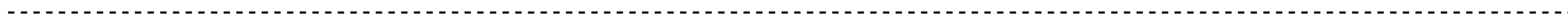

Input  
Myo

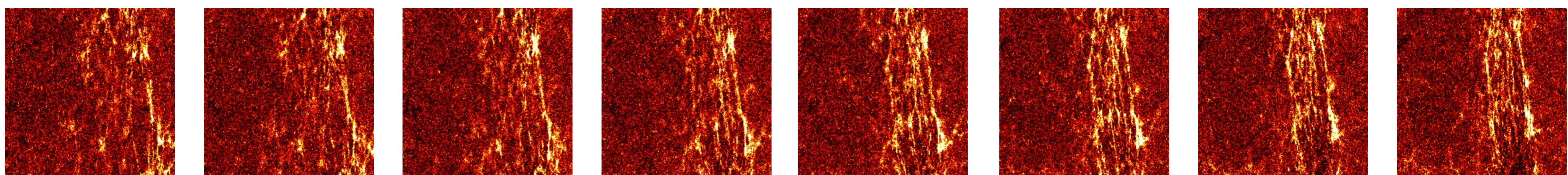

GT  
Jub

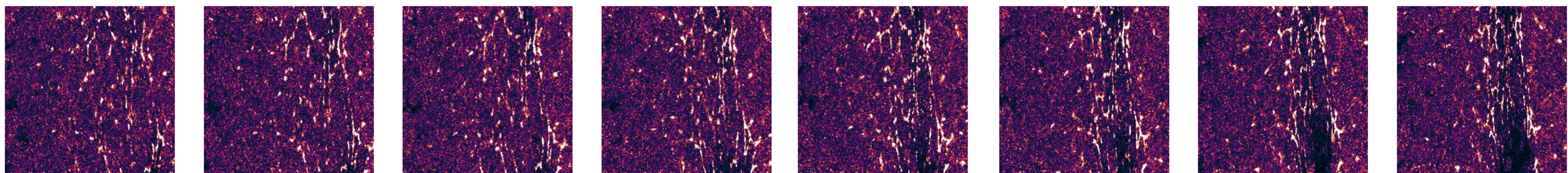

Our  
Jub

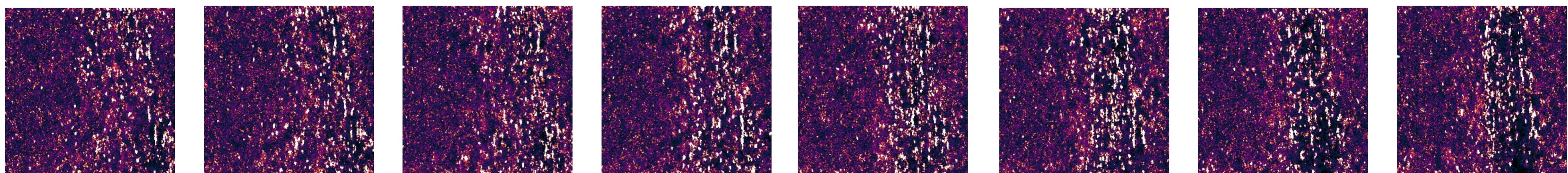

Pix2Pix  
Jub

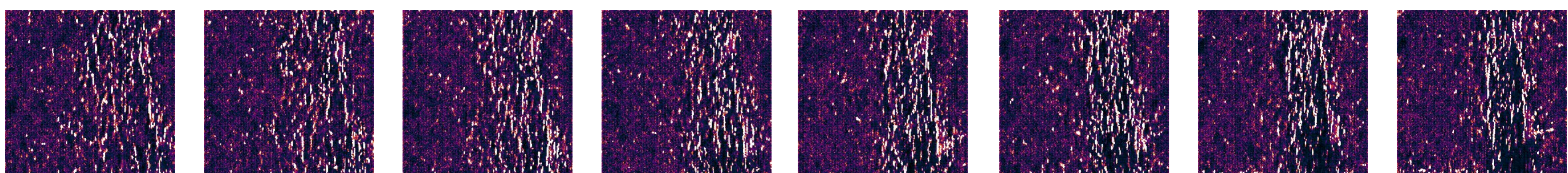

Supplement: btac719_Supplementary_Data [file btac719_supplementary_data.zip › figures/spp_more_PLP_myo-aju.drawio.pdf]

$t=20$

$t=25$

(a)

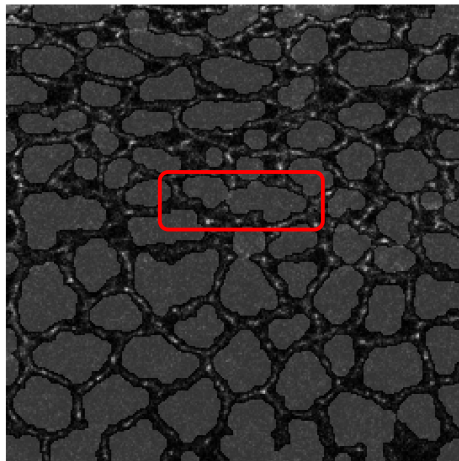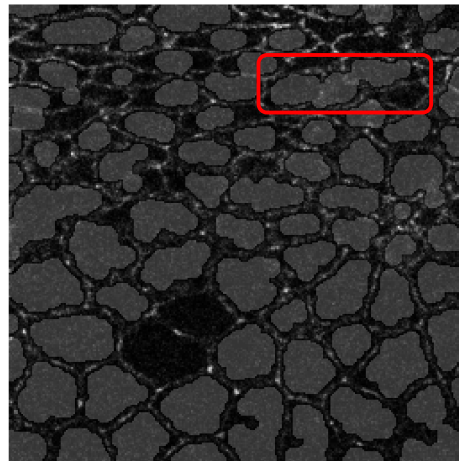

(b)

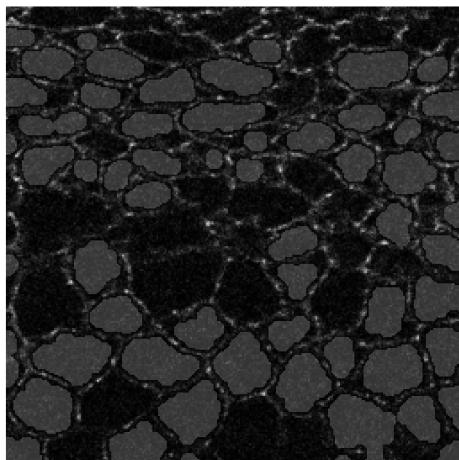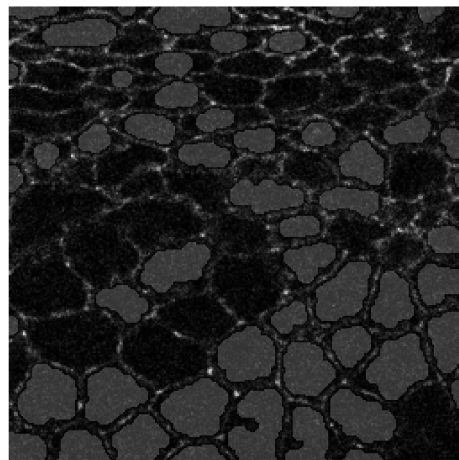

Supplement: btac719_Supplementary_Data [file btac719_supplementary_data.zip › figures/spp1_1.drawio.pdf]

$t \rightarrow$

cell 1

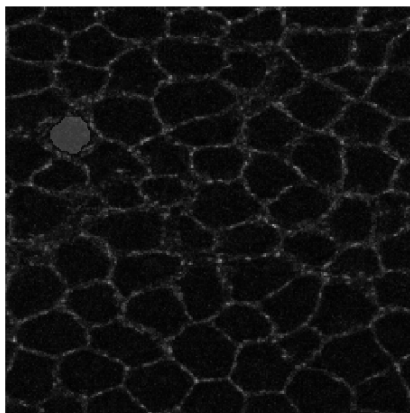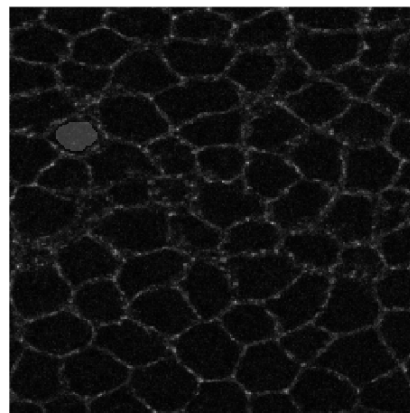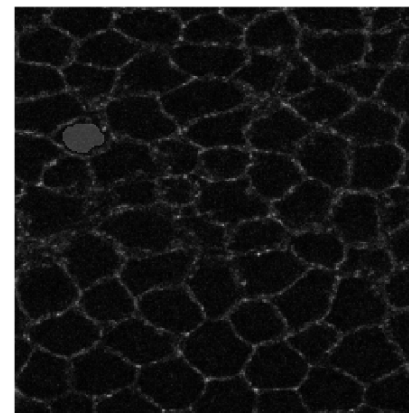

cell 2

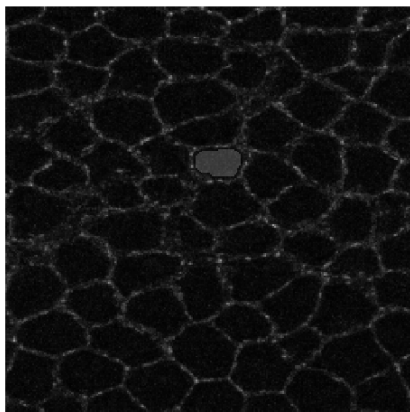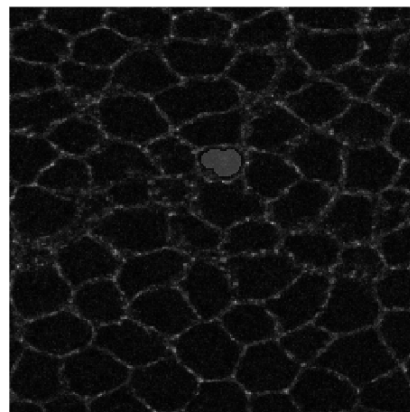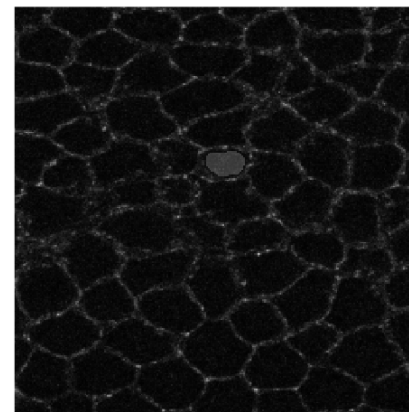

cell 3

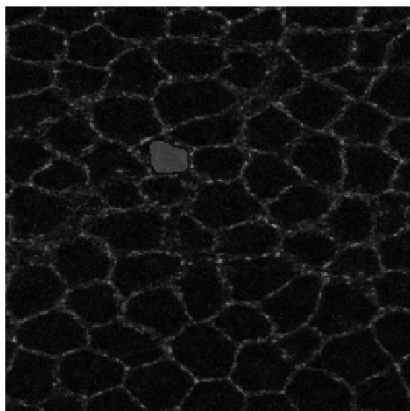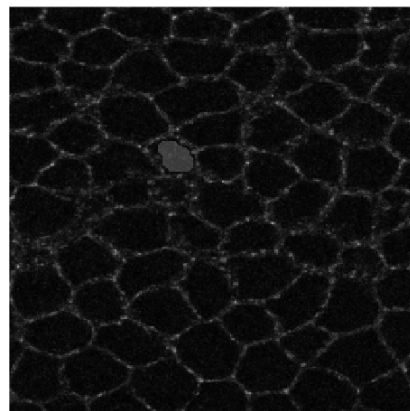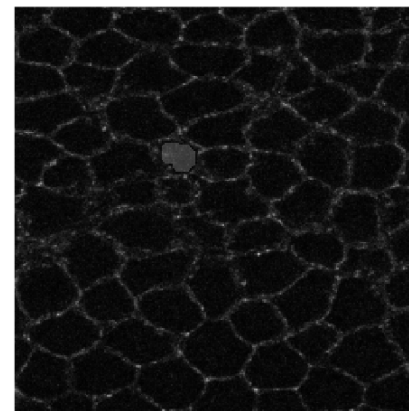

Supplement: btac719_Supplementary_Data [file btac719_supplementary_data.zip › figures/spp2_1.drawio.pdf]

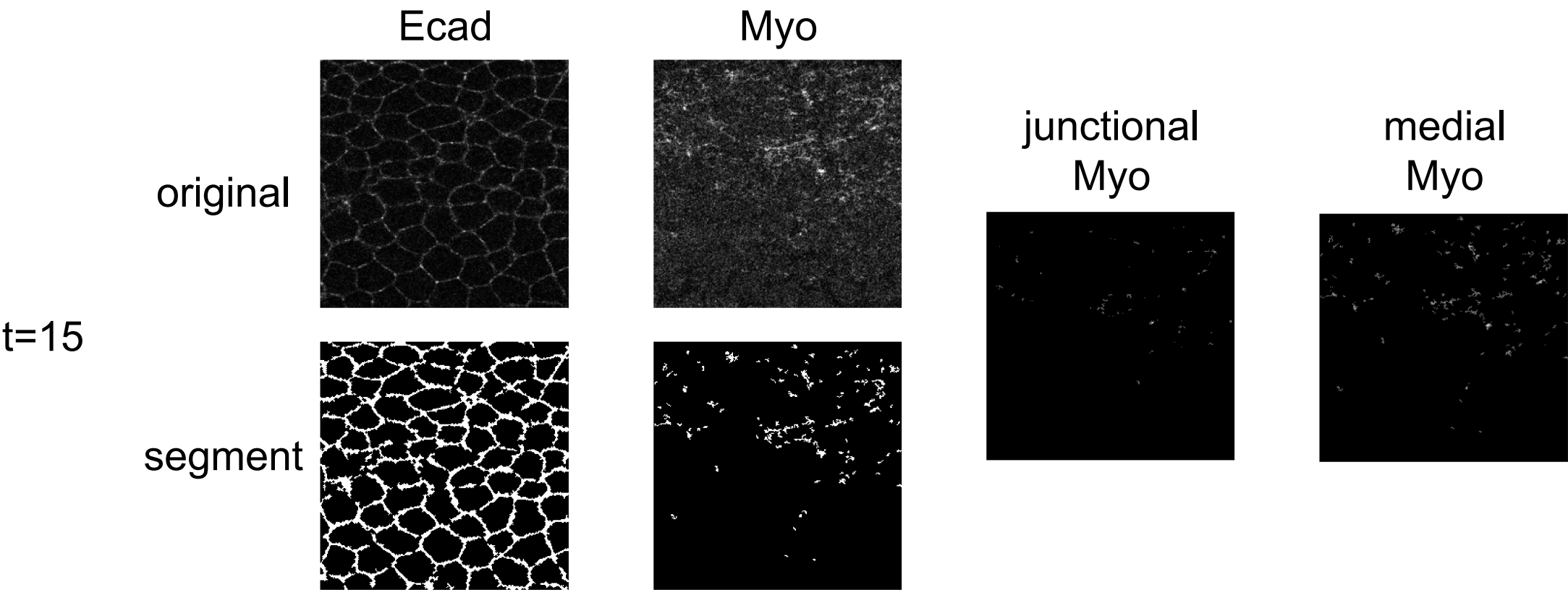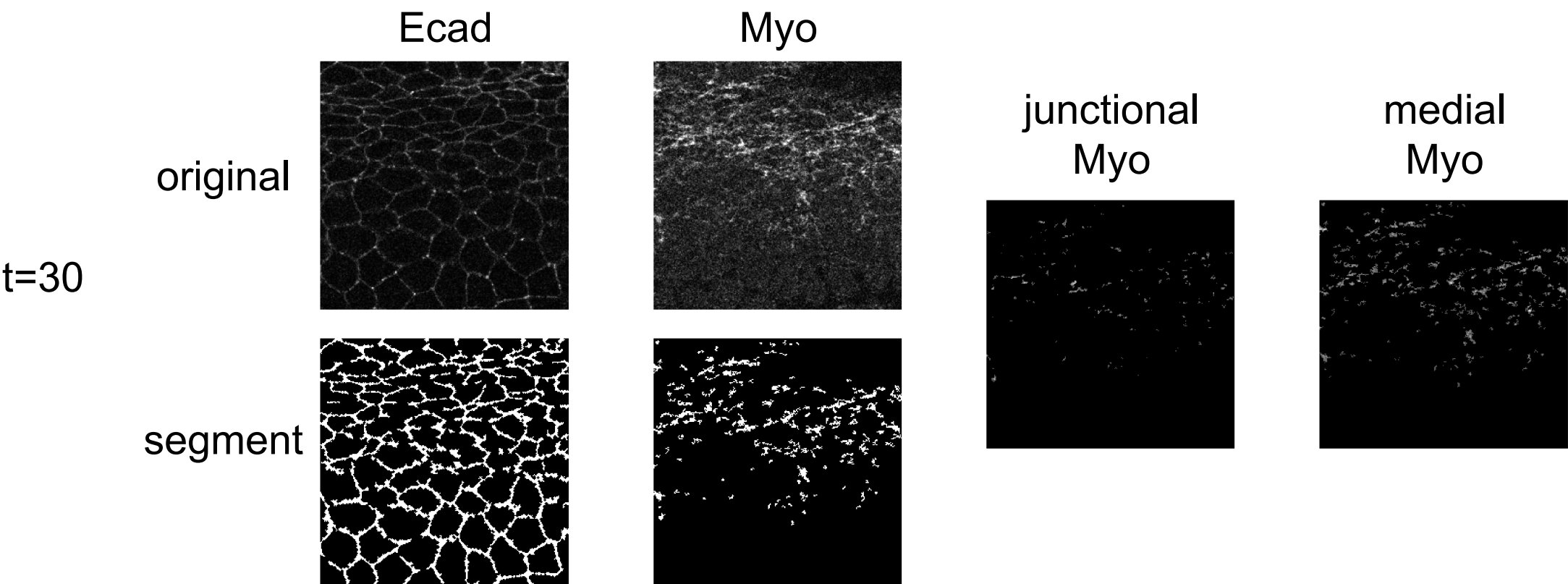

Supplement: btac719_Supplementary_Data [file btac719_supplementary_data.zip › figures/spp3_1.drawio.pdf]

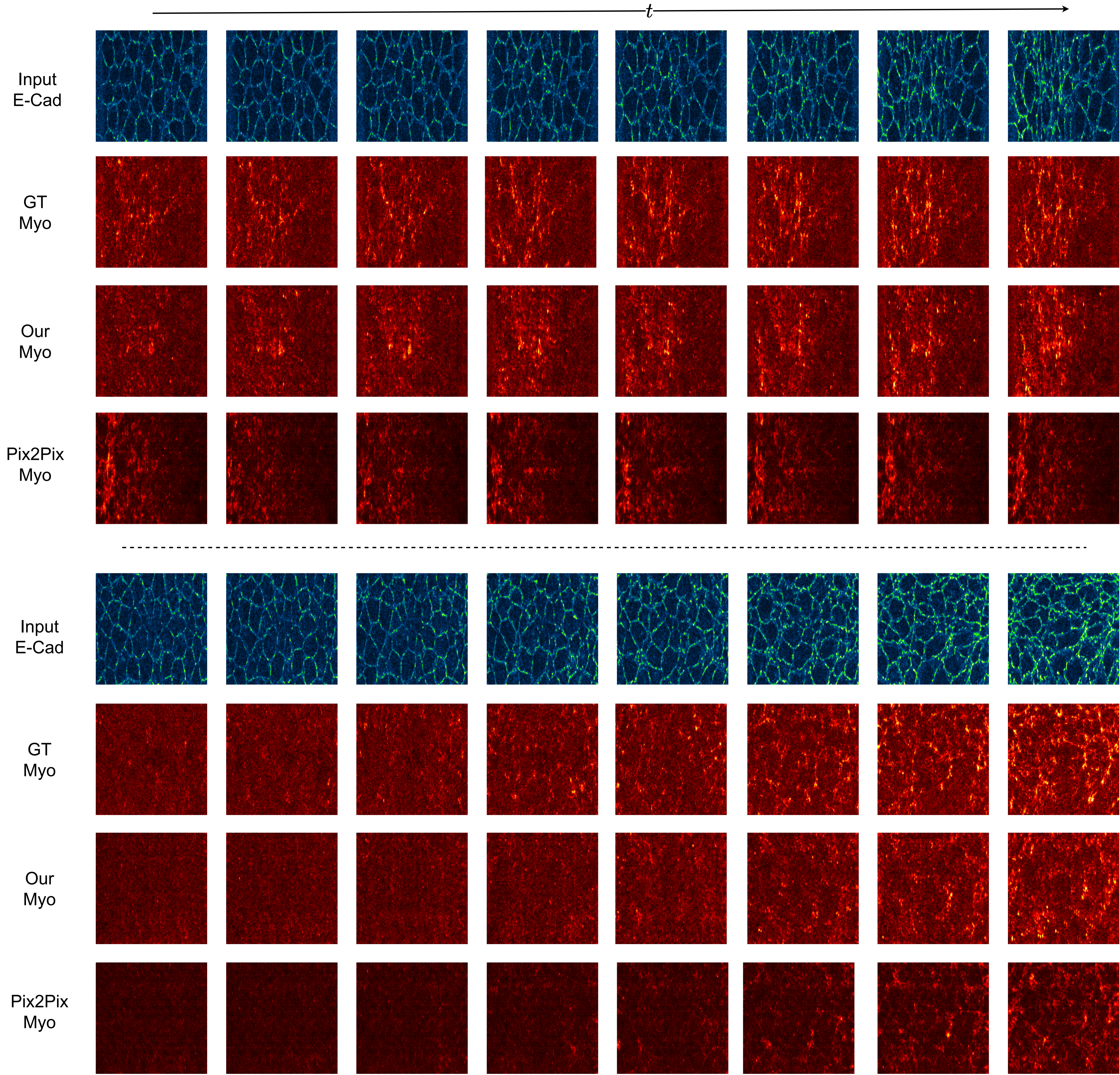

Supplement: btac719_Supplementary_Data [file btac719_supplementary_data.zip › figures/spp_more_PLP_ecad-myo.drawio.pdf]
